# A depauperate immune repertoire precedes evolution of sociality in bees

**DOI:** 10.1186/s13059-015-0628-y

**Published:** 2015-04-24

**Authors:** Seth M Barribeau, Ben M Sadd, Louis du Plessis, Mark JF Brown, Severine D Buechel, Kaat Cappelle, James C Carolan, Olivier Christiaens, Thomas J Colgan, Silvio Erler, Jay Evans, Sophie Helbing, Elke Karaus, H Michael G Lattorff, Monika Marxer, Ivan Meeus, Kathrin Näpflin, Jinzhi Niu, Regula Schmid-Hempel, Guy Smagghe, Robert M Waterhouse, Na Yu, Evgeny M Zdobnov, Paul Schmid-Hempel

**Affiliations:** Experimental Ecology, Institute of Integrative Biology, ETH Zürich, CH-8092 Zürich, Switzerland; Department of Biology, East Carolina University, Greenville, NC 27858 USA; School of Biological Sciences, Illinois State University, Normal, IL 61790 USA; Theoretical Biology, Institute of Integrative Biology, ETH Zürich, CH-8092 Zürich, Switzerland; Computational Evolution, Department of Biosystems Science and Evolution, ETH Zürich, 4058 Basel, Switzerland; Swiss Institute of Bioinformatics, 1211 Lausanne, Switzerland; School of Biological Sciences, Royal Holloway University of London, London, TW20 0EX UK; Department of Crop Protection, Faculty of Bioscience Engineering, Ghent University, 9000 Ghent, Belgium; Maynooth University Department of Biology, Maynooth University, Maynooth, Kildare, Ireland; Department of Zoology, School of Natural Sciences, Trinity College Dublin, Dublin, 2 Ireland; School of Biological and Chemical Sciences, Queen Mary University of London, E1 41NS London, UK; Department of Apiculture and Sericulture, University of Agricultural Sciences and Veterinary Medicine Cluj-Napoca, Cluj-Napoca, 400372 Romania; Institut für Biologie, Molekulare Ökologie, Martin-Luther-Universität Halle-Wittenberg, Wittenberg, 06120 Germany; USDA-ARS Bee Research Laboratory, Beltsville, MD 20705 USA; German Centre for Integrative Biodiversity Research (iDiv) Halle-Jena-Leipzig, 04103 Leipzig, Germany; Institut für Biologie, Tierphysiologie, Martin-Luther-Universität Halle-Wittenberg, Wittenberg, 06099 Germany; College of Plant Protection, Southwest University, Chongqing, 400716 PR China; Department of Genetic Medicine and Development, University of Geneva Medical School, 1211 Geneva, Switzerland; Computer Science and Artificial Intelligence Laboratory, Massachusetts Institute of Technology, Cambridge, MA 02139 USA; The Broad Institute of MIT and Harvard, Cambridge, MA 02142 USA

## Abstract

**Background:**

Sociality has many rewards, but can also be dangerous, as high population density and low genetic diversity, common in social insects, is ideal for parasite transmission. Despite this risk, honeybees and other sequenced social insects have far fewer canonical immune genes relative to solitary insects. Social protection from infection, including behavioral responses, may explain this depauperate immune repertoire. Here, based on full genome sequences, we describe the immune repertoire of two ecologically and commercially important bumblebee species that diverged approximately 18 million years ago, the North American *Bombus impatiens* and European *Bombus terrestris*.

**Results:**

We find that the immune systems of these bumblebees, two species of honeybee, and a solitary leafcutting bee, are strikingly similar. Transcriptional assays confirm the expression of many of these genes in an immunological context and more strongly in young queens than males, affirming Bateman’s principle of greater investment in female immunity. We find evidence of positive selection in genes encoding antiviral responses, components of the Toll and JAK/STAT pathways, and serine protease inhibitors in both social and solitary bees. Finally, we detect many genes across pathways that differ in selection between bumblebees and honeybees, or between the social and solitary clades.

**Conclusions:**

The similarity in immune complement across a gradient of sociality suggests that a reduced immune repertoire predates the evolution of sociality in bees. The differences in selection on immune genes likely reflect divergent pressures exerted by parasites across social contexts.

**Electronic supplementary material:**

The online version of this article (doi:10.1186/s13059-015-0628-y) contains supplementary material, which is available to authorized users.

## Background

Group living confers many benefits (for some examples see [1-4]) and highly social insects such as ants - epitomes of a highly organized animal society - have risen to ecological dominance in many ecosystems of the world [5]. But group living is also associated with costs. Parasites present an enhanced risk to social animals, as large group size [6], high density, and often close relatedness among individuals increases the exposure and spread of infectious diseases (for example, [7-14]; but see [15]). On the continuum of sociality, eusocial insects are an extreme, forming dense colonies with often very highly related individuals (up to an average coefficient of relatedness of *r* = 0.75), where individuals perform specific functions within the group, at its simplest specializing as reproductive and worker castes. Given a generally higher risk of disease in social insect colonies, it is surprising that complete genome sequencing revealed that honeybees (*Apis mellifera*) had approximately only one-third as many immune genes as the two existing genomic model insect systems at the time, *Drosophila melanogaster* and *Anopheles gambiae* [16]. Honeybee biology differs from these model species in several ways, which may partly explain the striking difference in immune genome organization among these taxa. For instance, honeybees have a suite of hygienic behaviors where they groom both themselves and others, and live on food (pollen and nectar) that is also relatively clean (not withstanding the fact that food-borne diseases have been described in honeybees, for example, [17,18]). The observation that ant genomes also have few immune genes [19] indicates that this deficiency may be a more general characteristic of social hymenoptera and not primarily an artifact of honeybee breeding [20]. Sociality may instead typically allow for group-based defenses ('social immunity' [21]) that should reduce selective pressures on the evolution and maintenance of immune genes. Given the recent and dramatic declines in populations of important bee pollinators [22-24] and the role of parasites in some of these declines (for example, [23,25,26]), understanding the architecture of the immune system of bees in relation to other insects is increasingly important.

Bumblebees (genus *Bombus*) are essential natural and commercial pollinators and have been declining due to anthropogenic disturbances, including habitat destruction and fragmentation (reviewed in [24,27]), but also due to introduced competitors [28,29], and more recently pesticides [30,31] and parasites [24,26,32,33] have been implicated as important drivers of declines. Bumblebee declines are of both ecological and practical importance as they contribute substantially to human food crops either directly [24,27,34] or as part of a community of wild pollinators that are supplemented by managed honeybees [35]; therefore, they also aid the maintenance of plant diversity [36]. Among *Bombus* species, *Bombus impatiens*, and *Bombus terrestris*, both key commercial and natural pollinators, have been most extensively studied, in particular for host-parasite interactions [37-40]. These two species occupy comparable niches in North America (*B. impatiens*) and Europe (*B. terrestris*). They last shared a common ancestor approximately 18 million years ago [41].

While *B. terrestris* and *B. impatiens* share ecological factors, such as diet, with honeybees, they differ from the latter in colony organization, sociality, longevity, and mating system. Bumblebees, including *B. terrestris* and *B. impatiens*, are less advanced in their sociality than honeybees, as the physiological and morphological difference between queens and workers is not as pronounced, division of labor is weak, and colonies are much smaller (dozens or hundreds instead of thousands of workers) and very simply organized [42]. Bumblebee colonies as a whole are also shorter-lived than those of honeybees, with bumblebee queens living for one year but the colony persisting for only a few months, whereas honeybee queens and their colonies can live for several years. Like most bumblebee species, *B. terrestris* queens mate singly and *B. impatiens* queens mate singly or occasionally doubly [43], whereas *Apis* queens mate with between 10 and over 100 males [44-47]. This has important consequences for disease susceptibility as both multiply mated honeybees [48] and *B. terrestris* [49] that were artificially inseminated with sperm from multiple males produce colonies with lower parasite loads than colonies from singly mated queens.

All of these differences may have profound consequences for the evolution of their immune systems. Here, using the recently sequenced complete genomes of both the North American *B. impatiens* and the European *B. terrestris* we explore patterns of immune system evolution across a social gradient by comparing the immune repertoire and sequences of immune genes of these two species of bumblebees with those of two species of highly social honeybees and the solitary leaf-cutting bee *Megachile rotundata*.

## Results

### Immunological repertoire

**Figure 1:**
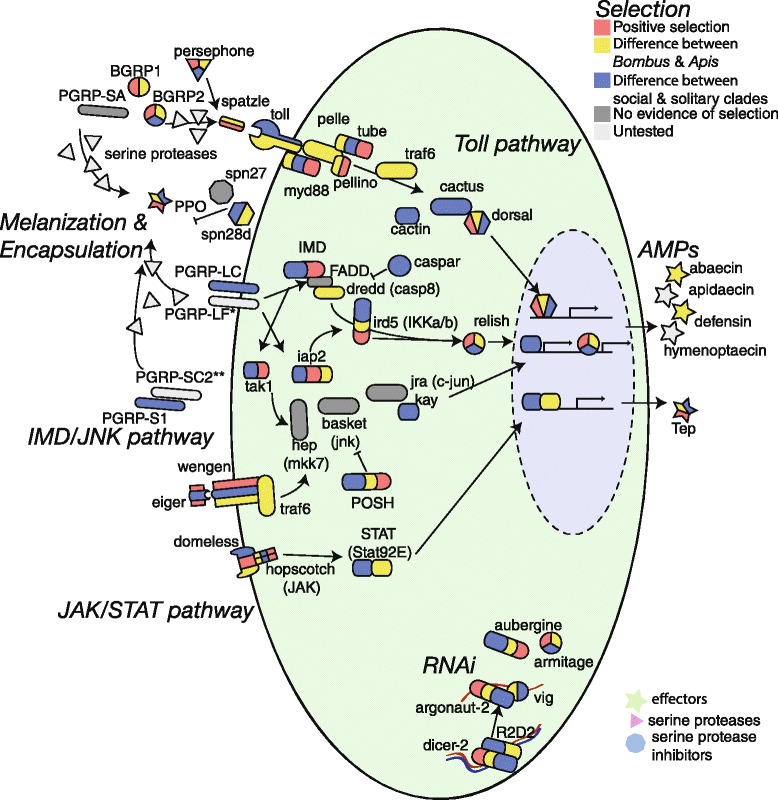
Diagram of the classical insect immune responses to parasites: *Toll*, *IMD*/*JNK*, *JAK/STAT* pathways and the melanization and antiviral RNA interference responses. Colors of the genes indicate evidence of selection as detected by either positive selection (across the four taxa phylogeny, on the branch between *Bombus* and *Apis*, the branch leading to *Bombus*, *Apis*, or *Megachile*) in red, or differences in selection between *Bombus* and *Apis* (yellow), or between the social and solitary clades (blue). More complete information about selection on these genes can be found in Additional files 8, 9, 10 and 11. *PGRP-LF is only found in *B. impatiens*. **PGRP-SC2 is not among the automated predictions for *B. terrestris*, although sequence in the trace archive suggests that it is present. We also detect expression of PGRP-SC2 in *B. terrestris*. AMP, anti-microbial peptide.

**Figure 2:**
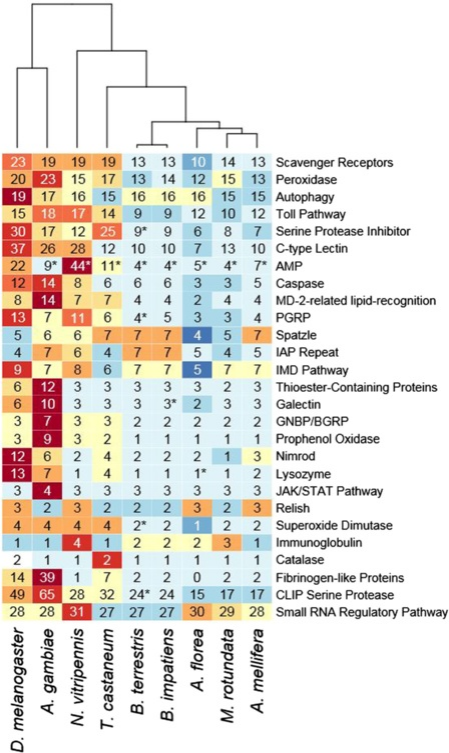
Number of genes belonging to 27 families of immune genes from OrthoDB. The colors in this heatmap reflect the number of genes in that category relative to the other species. Numbers with asterisks were manually adjusted according to our annotation efforts or the literature. The tree represents a clustering analysis using Euclidean distances based on the number of genes within these groups.

**Figure 3:**
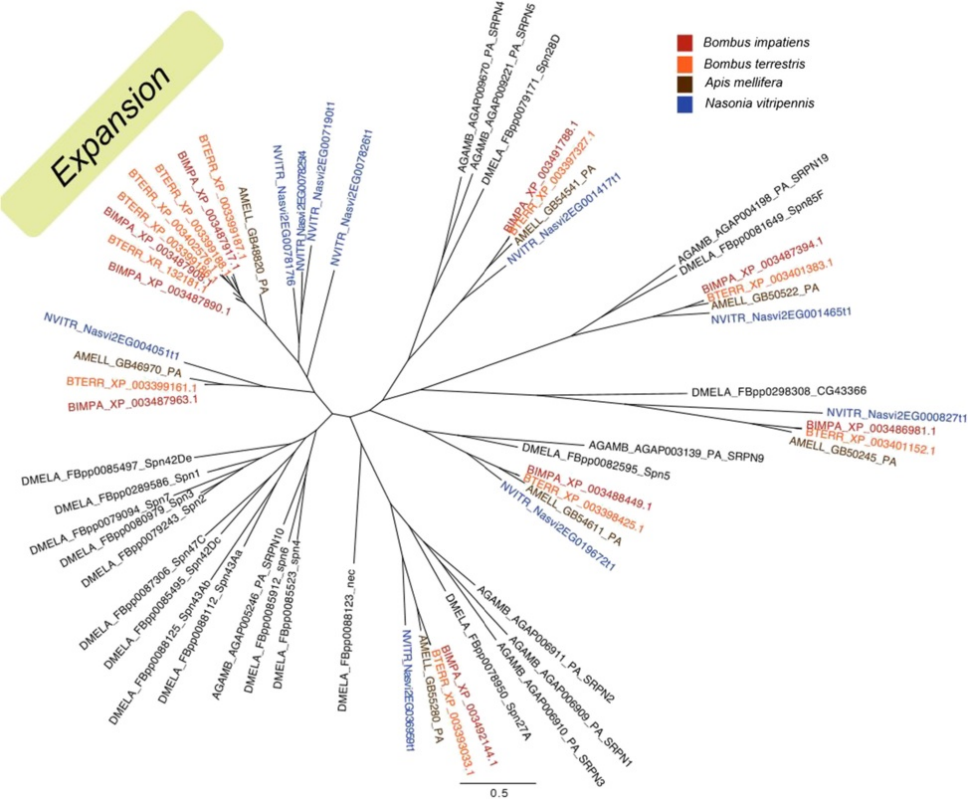
Gene tree of serine protease inhibitors showing the expansion within *Bombus* (green box). Hymenopteran species are labeled by color and Dipterans are labeled black.

Regardless of social organization, all bee species examined shared a core set of immune genes, including all members of the canonical immune pathways (Figure 1) with only minor differences in gene numbers (Figure 2). We found no relationship between the degree of sociality and the total number of canonical immune-related genes. With regard to important immune response effectors, such as anti-microbial peptides (AMPs), both *Bombus* species have only a single copy of *defensin*, which is present in two copies in *A. mellifera*; on the other hand, *Bombus* have an expanded set of serine protease inhibitors (serpins; Figure 3). We identified five, highly similar (average 75% sequence similarity), putative serpin 3/4-like genes in *B. terrestris*. Initial homology searches found four serpins (XP_003399186.1, XP_003399187.1, XP_003399188.1, XP_003402576.1) while a revised search using proteomic data confirmed the expression of a fifth serpin, originally described as a pseudogene (XR_132181.1; Figure 3). The proteomic data also identified two unique multiple-peptide supported isoforms of XR_132181.1 (TJ Colgan *et al*., unpublished data). Four serpins are clustered on genomic scaffold 11.4 while the fifth serpin (XP_003402576.1) is on an unassembled genome contig (GroupUn430). *B. impatiens* appears to have three novel serpins (XP_003487908.1, XP_003487890.1, and XP_003487917.1) clustered on genomic scaffold scf_0203. Homology searches for bumblebee serpins against sequences of other members of the superfamily Apoidea identified single orthologs for the eusocial honeybee *A. mellifera*, and the solitary leafcutter bee *M. rotundata*. Outside of the Hymenoptera, these serpins shared sequence similarity with serpin-1 (alaserpin) of the lepidopteran *Manduca sexta*.

**Figure 4:**
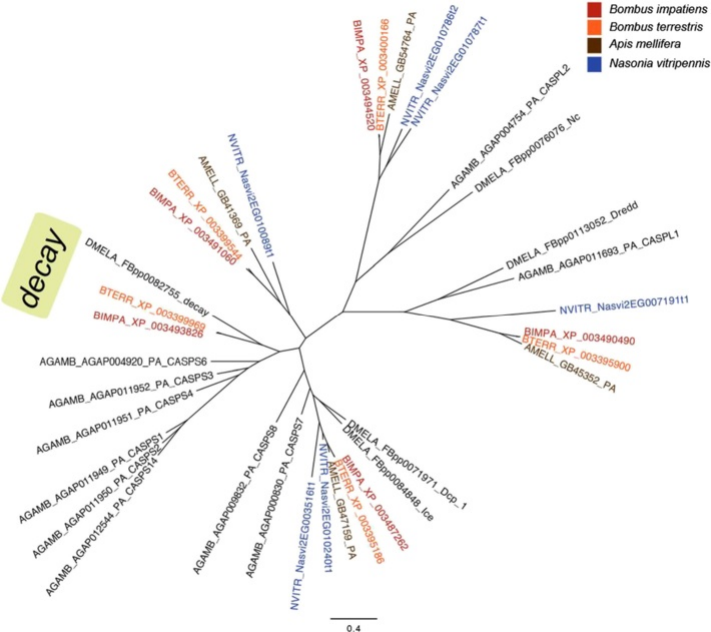
Gene tree of caspases showing the *Bombus* genes that appear similar to *D. melanogaster decay* (green box). Hymenopteran species are labeled by color and Dipterans are labeled black.

We also find what appears to be a homolog of the apoptosis-involved caspase *decay* (Figure 4). There also appears to be a Hymenoptera-specific clade of caspases that share the most homology with *Ice* in *Drosophila*. We find an additional PGRP (peptidoglycan receptor protein) in *B. impatiens* (XP_003487752), which is missing in *B. terrestris* and *A. mellifera*. On the genomic sequence, this novel PGRP is immediately downstream of XP_003487751, which is homologous to XP_003400160 in *B. terrestris* and XP_392452 in *A. mellifera*, likely from tandem duplication*.*

### Immunological expression

**Figure 5:**
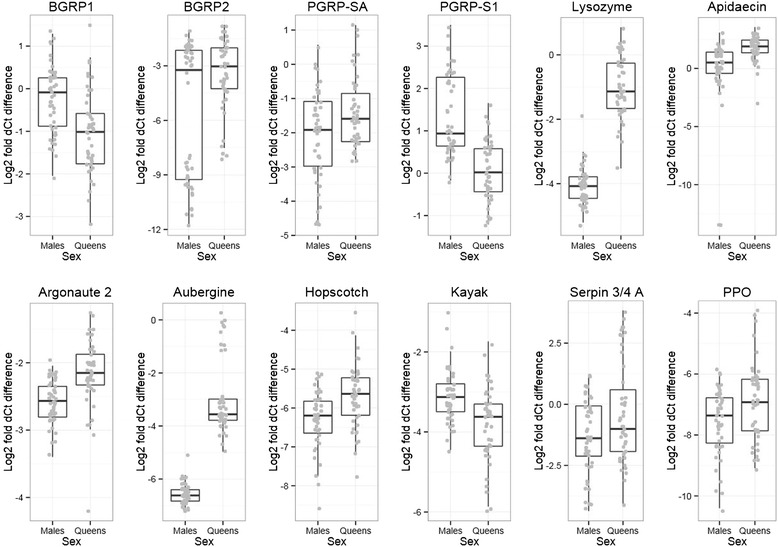
Logfold gene expression relative to invariant housekeeping genes in males and young queens (gynes). All genes shown here are significantly differentially expressed between the sexes. Full details of these statistics can be found in the supplemental materials.

**Figure 6:**
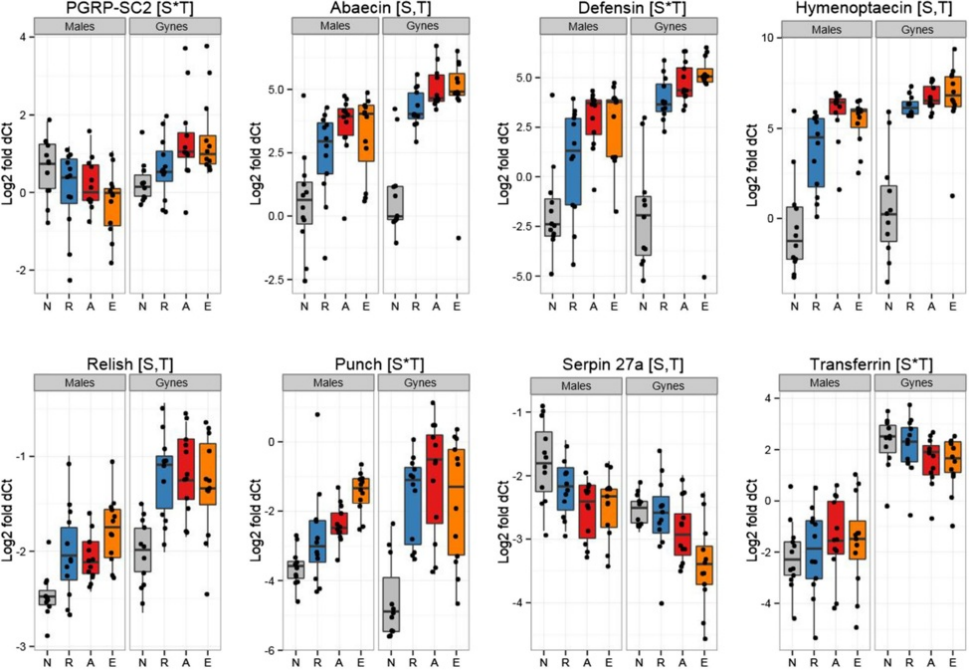
Logfold gene expression relative to invariant housekeeping genes in males and gynes according to treatment (x-axis: N, naïve; R, Ringer injection; A, *Arthrobacter globiformis* injection; E, *Escherichia coli* injection). Next to the gene name we depict whether the expression differed significantly according to sex (S), treatment (T), or the interaction between sex and treatment (S*T). Full details of these statistics can be found in the supplemental materials.

We used quantitative PCR to determine whether 27 candidate immune genes (Table 1) were functionally expressed in *B. terrestris*, including if they were differentially expressed following exposure to Gram-negative or Gram-positive bacterial cues. We measured expression in gynes and males also to investigate sex-specific patterns. All surveyed genes were actively expressed in both gyne and male *B. terrestris*, including the novel serpins (*serpin 3*/*4 A*, *serpin 3*/*4 B*, *alaserpin*) and the *decay* homolog. Both sex (Tables 2 and 3; Figure 5) and treatment (Figure 6) significantly influenced expression of this battery of genes and the different sexes responded differently to the treatments as revealed by the sex*treatment interaction (Tables 2 and 3; Figure 6). The recognition receptors *beta-glucan receptor protein 2* (*BGRP2*) and *peptidoglycan receptor protein SA* (*PGRP-SA*) were more strongly expressed in queens than males, but *BGRP1* and *PGRP-LB* had male-biased expression. *PGRP-SC2* was more strongly expressed in queens but was also upregulated in queens given the challenge whereas males downregulated this gene upon challenge. All antimicrobial peptides (AMPs) were more strongly expressed in queens than males and most were induced upon challenge and induced more dramatically in queens. The effectors *lysozyme*, *transferrin*, the signaling transducer *relish*, antiviral genes *argonaute* and *aubergine*, and melanization related genes *PPO* and *punch* follow a similar pattern with queen-biased expression and greater induction of expression when there was a significant treatment by sex interaction. An exception to this general pattern is the *serpin 27a*, which inhibits melanization. Queens had lower expression of this gene and the expression appears to be reduced upon bacterial exposure. Males did not reduce their expression of *serpin 27a* as intensely as the queens did.

**Table 1:** **Gene and primer details used for quantitative PCR**

**Gene**	**Putative gene function**	**NCBI accession**	**Forward primer**	**Reverse primer**	**Product size (base pairs)**
*AK*	Arginine kinase, housekeeping	AF_492888	CTGGACTCTGGTGTCGGTAT	GTCTTTTGGTGGATGCTTGT	129
*PLA2*	Phospholipase A2, housekeeping	FN_391388	TATCTTTCAATGCCCAGGAG	GTCGTAACAAATGTCATGCG	129
*ef1α*	Elongation factor 1α	XM_003401944	GCTGGTGACTCGAAGAACAATC	GGGTGGTTCAACACAATAACCTG	74
*BGRP1*	Recognition receptor, Toll pathway	XM_003397996	AACGTGGAAGTCAAAGATGG	GCGAACGATGACTTGGTATT	206
*BGRP2*	Recognition receptor, Toll pathway	XM_003394713	TAACTCCCTTTGGAAACACG	GGCGGTAAAATACTGAACGA	249
*PGRP-S1*	Recognition receptor, Imd pathway	XM_003400112	TTTCCATGTTGCTCGCTTCG	CGCGGTTTCCCTTTCGATATTAG	77
*PGRP-LC*	Recognition receptor, Imd pathway	XM_003396463	CAGCCACCTACGACAGATTT	GTACATTCCGCTTGTGTCCT	101
*PGRP-SA*	Recognition receptor, Toll pathway	XM_003401893	CGTGAAGGAGCTCATACCAT	CCAGGACTCATAGTGGCTGT	200
*PGRP-SC2*	Recognition receptor, Imd pathway	XM_003493213	TTGGTTGGCGAAGATGGAAAC	CGCGCTTGGATTATGACCAAC	132
*pelle*	Signal molecule, Toll pathway	XM_003399470	TAAATCGACCTATGCAAGCC	GGGTATAGCTGCTTCTGCTG	107
*relish*	Signal molecule, Imd pathway	XM_003399472	CAGCAGTAAAAATCCCCGAC	CAGCACGAATAAGTGAACATA	156
*basket*	Signal molecule, JNK pathway	XM_003402794	GGAACAAGATAATCGAGCAACTG	CTGGCTTTCAATCGGTTGTG	177
*hopscotch*	Signal molecule, JAK/STAT pathway	XM_003401903	CACAGACTGAAGCAGGTTGA	CATATGGGTAATTTGGTGCC	353
*abaecin*	Antimicrobial peptide (AMP)	XM_003394653	GCCACAATATGTGGAATCCT	ATGACCAGGGTTTGGTAATG	141
*apidaecin*	Antimicrobial peptide (AMP)	XM_003402966	CCCGACTAATGTACCTGCCA	GAAGGTGCGAATGTGTTGGA	131
*defensin*	Antimicrobial peptide (AMP)	XM_003395924	GTCTGCCTTTGTCGCAAGAC	GACATTAGTCGCGTCTTCTTCG	139
*hymenoptaecin*	Antimicrobial peptide (AMP)	XR_132450	TTCATCGTACTGGCTCTCTTCTG	AGCCGTAGTATTCTTCCACAGC	85
*TEPA*	Effector molecule, JAK/STAT pathway	XM_003399699	GCGTTCTATGACCACCTGTT	TACAGGTTACTCCACAGCCC	212
*lysozyme3*	Bacteriolytic effector	XM_003394052	TATGGGCAAGAAGATTCGAC	GTGTACATCGTTCACGCATC	219
*transferrin*	Iron-binding protein, antibacterial	XM_003401163	CAATTTCTTCACCGCATCCT	CCTCGTTATTTGGCTTGCAT	131
*ferritin*	Iron transportation protein	XM_003393332	AAAGAATTGGACGCAAATGG	CAGCGAACTGATGTCCAAGA	259
*serpin27a*	Serine protease inhibitor, prophenoloxidase cascade	XM_003392985	CCGATCATCCATTCGTATTC	ACCTGCACTTGATATCCCTG	164
*PPO*	Prophenoloxidase, melanin synthesis	HM142999	AGCGGCATAATACGTTGTGT	CCGAGGGATAGAAAGTCTCC	329
*punch*	Enzyme, melanin synthesis	XR_131852	ATTGCCAGGACACTTTCAAC	TACAAGCTGGAAACGGAAAC	211
*kayak*	JNK pathway	Bter:08277927	ACGCAATATGGGTGGCAGAA	TGAACGAAGACGACAGACCG	271
*serpin 3/4A*	Novel serine protease inhibitor	XM_003399138	GCAGAGACAAATGTTGAAGCAC	CACAGTCTGGGATAATGAAGAACC	78
*serpin 3/4B*	Novel serine protease inhibitor	XM_003399140	ATGGTGCTTTGTTCATCAGTCG	GACCCAATGACAGCAGTAACAG	97
*alaserpin*	Novel serine protease inhibitor	XM_003399139	TGCTGAAATGCTAGATGACACG	GCATATCGCTCGTTAACTCAGG	104
*argonaute 2*	RNA-interference, possible antiviral function	XM_003398481	AATTGCAAGATCAACCTGCC	CCTACCCAAAGACAAGGCAA	175
*aubergine*	RNA-interference, possible antiviral function	XM_003400641/XM_003400642	GTCGCCCTTCTGCATATCTC	AAGATCGAACTGCTATCCGC	190
*decay*	Caspase mediating apoptosis	XM_003399921	AAGAAGACCTCGGTCCTTAGAC	CAGCTGCAAATGAAGTAATGCG	74

**Table 2:** **MANOVA results for all validated immune genes**

	**Df**	**Pillai**	**Approximate F**	**Num Df**	**Den Df**	***P***
Sex	1	0.986	128.635	27	51	<0.0001
Treatment	3	1.445	1.823	81	159	0.00067
Sex*Treatment	3	1.365	1.640	81	159	0.0042
Residuals	77

**Table 3:** **Univariate ANOVA results for each gene tested in the MANOVA**

	**Sex**	**Treatment**	Sex*Treatment
*BGRP1*	***	~	–
*BGRP2*	***	~	–
*PGRP-S1*	***	–	–
*PGRP-LC*	–	–	–
*PGRP-SA*	**	–	–
*PGRP-SC2*	***	–	**
*abaecin*	***	***	~
*apidaecin*	***	~	–
*defensin*	***	***	*
*hymenoptaecin*	***	***	–
*lysozyme*	***	–	–
*transferrin*	***	–	*
*ferritin*	–	–	–
*tepA*	–	–	~
*relish*	***	***	–
*basket*	–	–	–
*hopscotch*	***	–	–
*kayak*	***	–	–
*punch*	–	***	*
*serpin ¾A*	**	–	–
*serpin ¾B*	–	–	–
*alaserpin*	–	–	–
*serpin27a*	***	***	–
*PPO*	*	–	–
*argonaute 2*	***	–	–
*aubergine*	***	–	–
*decay*	–	–	–

Transformed expression values (dCt) were treated as dependent on the sex of the bees (male/queen) and the treatment they received (naïve, sterile Ringers solution injection, injection with *Arthrobacter globiformis*, or injection with *Escherichia coli*). –, *P* > 0.1; ~, *P* < 0.1; *P < 0.05; **P < 0.01; ***P < 0.001; full statistics can be found in Additional file 13.

### Signatures of selection

**Figure 7:**
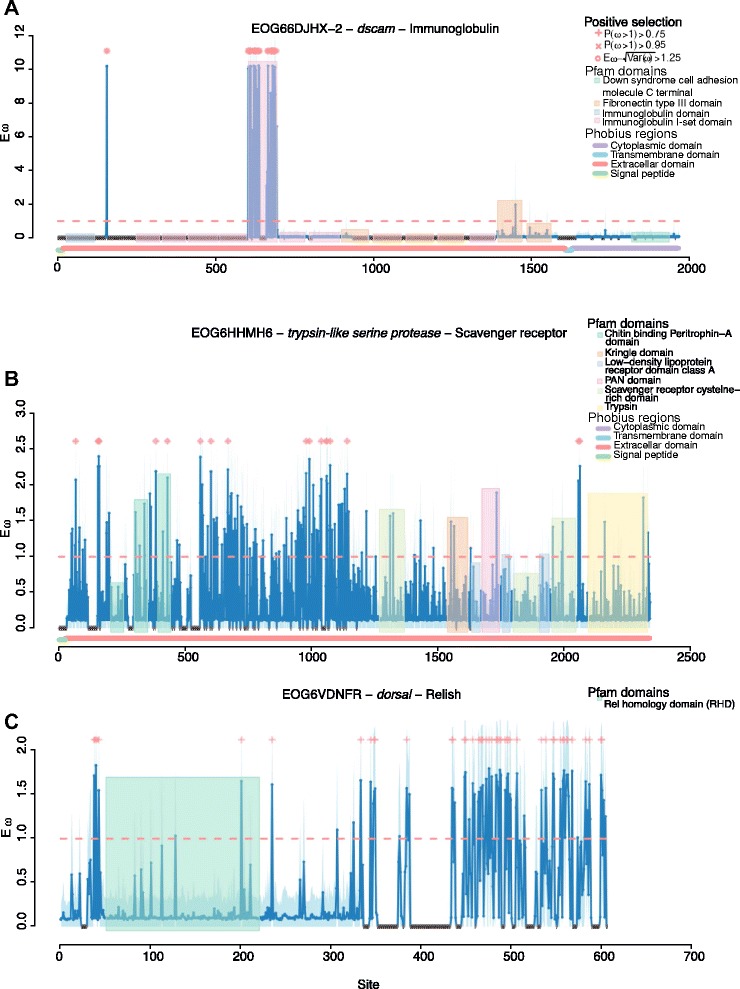
Sites under selection within the *Apis*, *Bombus* phylogeny for three genes of interest. The title for each gene presents the OrthoDB accession, the gene name, and the immune category. We only present a subset of the genes that showed an overall signature for selection highlighting codons at three different significance thresholds: Bayesian posterior probability >0.75 (plus signs along the top of each panel), >0.95 (x’s), and where Eω - sqrt(Var(ω)) > 1.25 (circles). The blue shadow indicates an estimate of error at each codon. We show Pfam domains in colored blocks and Phobius regions along the x-axis. Crosshatched regions were trimmed from the alignment.

**Figure 8:**
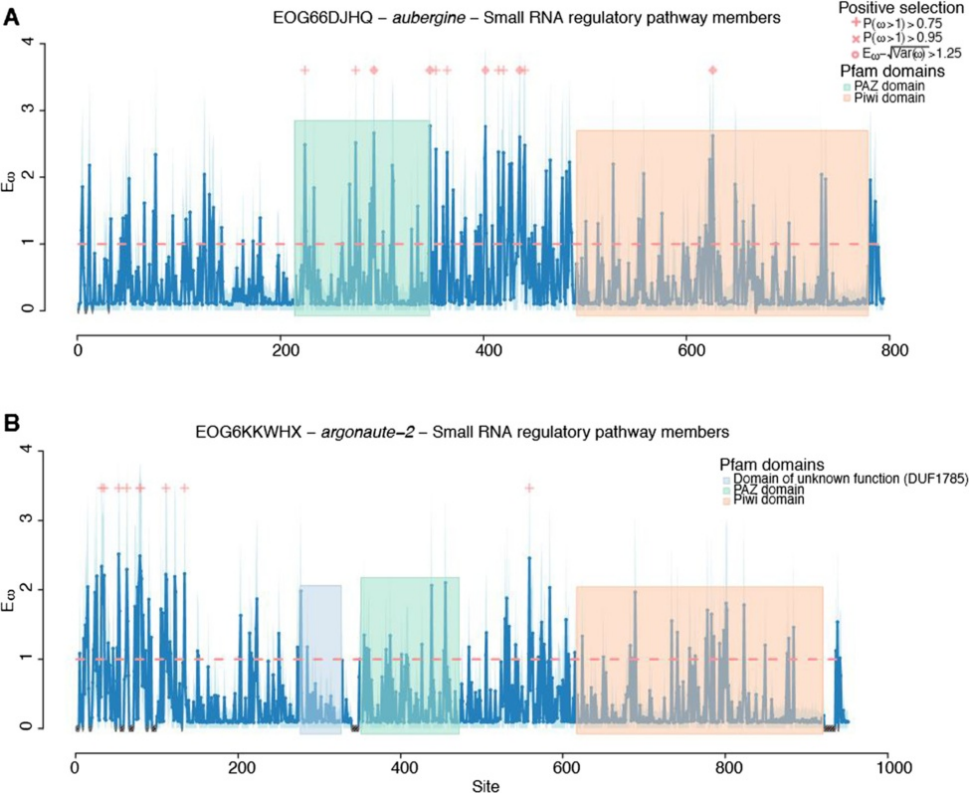
Sites under selection within the *Apis*, *Bombus* phylogeny for two viral response genes. The title for each gene presents the OrthoDB accession, the gene name, and the immune category. We only present a subset of the genes that showed an overall signature for selection highlighting codons at three different significance thresholds: Bayesian posterior probability >0.75 (plus signs along the top of each panel), >0.95 ('x's), and where Eω - sqrt(Var(ω)) > 1.25 (circles). The blue shadow indicates an estimate of error at each codon. We show Pfam domains in colored blocks and Phobius regions along the x-axis. Crosshatched regions were trimmed from the alignment.

**Figure 9:**
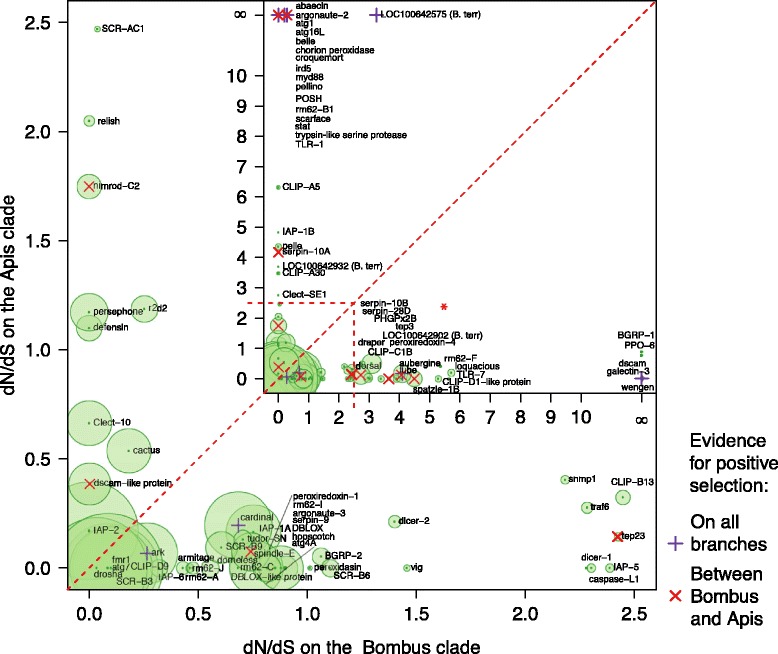
Differences in evolutionary pressure between *Apis* and *Bombus* across orthology groups. Names are taken from *D. melanogaster* when available. The size of the point is scaled according to the proportion of codons that are evolving under different selection in the two clades. Names were moved to improve legibility taking care to maintain x-axis position in the insert (denoted with an asterix). Full table of these results can be found in Additional file 8.

**Figure 10:**
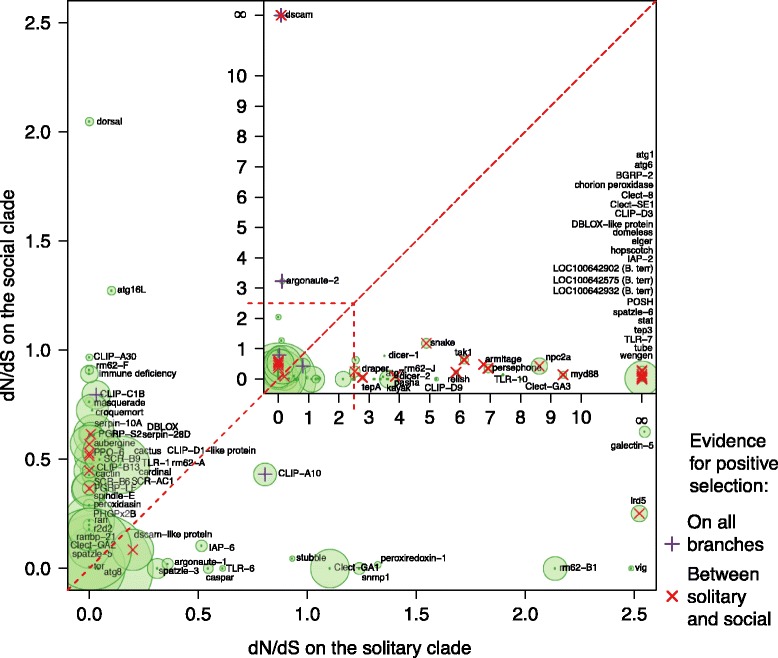
Differences in evolutionary pressure between social (*Apis* and *Bombus*) and solitary (*M. rotundata*) across orthology groups. Names are taken from *D. melanogaster* when available. The size of the point is scaled according to the proportion of codons that are evolving under different selection in the two clades Names were moved to improve legibility. A full table of these results can be found in Additional file 9.

While we did not identify any pattern of immune gene numbers varying with sociality, we did find variation in the evolution of these immune genes both between the highly social *Apis* clade and the less social *Bombus* clade, and between the solitary *Megachile* and the broader social clade containing *Apis* and *Bombus.* Globally, the ratio of non-synonymous to synonymous substitutions was ω = 0.12 ± 0.01 (mean ± standard error of the mean) and ω = 0.10 ± 0.01 in the four- and five-taxa M0 analyses, respectively (Additional files 1 and 2), although ω differed dramatically across ortholog groups (range 0.367 to 0.0001, 0.30 to 0.0047; Additional files 3, 4, 5, 6, and 7). The orthologs of *dscam* (down syndrome cell adhesion molecule), three antiviral proteins (*aubergine*, *argonaute 2*, and *rm62*) a trypsin-like serine protease that is homologous to the scavenger receptor *tequila*, a CLIP serine protease orthologous to CG4998, the peroxidase *cardinal*, a caspase *ark*, and the Toll signaling protein *pellino* showed evidence of positive selection across the four-taxa (*Apis* and *Bombus*) phylogeny (Table 4; Figures 7 and 8). Across the whole five-taxa phylogeny we again found evidence for positive selection on *argonaute 2*, *dscam*, *ark*, and the CG4998 ortholog, and additionally found positive selection on a second CLIP serine protease without a clear *D. melanogaster* ortholog but which is similar to CG11843 and *snake*, which are involved in *toll* signaling [50] (Table 5). In the branch leading to *Apis* the small RNA regulatory or anti-viral gene *drosha*, and the RNA helicase *rm62*, which has been implicated in both RNA interference [51] and bacterial response [52], the bacterial recognition gene *BGRP1*, a serine protease inhibitor, the caspase *ark* and *IMD* of the *IMD* pathway, are under selection (Table 6). On the branch leading to *Bombus* we find evidence of selection on *argonaute 2*, *rm62-F* (which is also an RNA helicase but has not been directly linked to immune responses), and the *toll-7* receptor, which has been implicated in viral defenses [53]. We also find evidence of selection on a number of members of the toll pathway, including *dorsal*, *myd88*, and *BGRP1*, which recognizes bacterial pathogens and initiates toll pathway signaling. *Domeless*, the receptor of the JAK/STAT pathway, had the most sites showing evidence of selection while *dorsal* showed stronger evidence of positive selection but across fewer sites. Two catalases, *ark* and *catalase*, a serpin and a scavenger receptor, *snmp1*, also appear to be under selection in bumblebees (Table 7; Figure 9). A number of genes show evidence of different selection between honeybees and bumblebees (Figure 9; Table 8; Additional file 8), including *dorsal*, *spaetzle*, and *tube*, all from the toll pathway, a nimrod gene, *argonaute 2*, a number of serpins, and *dscam.* Considerably more genes differ in selection between the social and solitary clades (Figure 10; Additional file 9) perhaps in part due to the difference in time since sharing an ancestor with both *Bombus* and *Apis*. However, genes that exhibit signs of different selection within the social clades (upper diagonal in Figure 10) are likely more robust than those showing signs of selection only in the solitary *M. rotundata* (lower diagonal) as the genes that appear to be evolving rapidly in the solitary group might be inflated due to the disproportionate phylogenetic distance of *M. rotundata* to the *Apis* and *Bombus* clades. A summary of genes for which we found evidence of selection and according to which selection model is provided in Additional file 10 (four taxa: *Bombus* and *Apis*) and Additional file 11 (five taxa: *Bombus*, *Apis*, and *Megachile*).

**Table 4:** **Genes under positive selection (using FDR < 0.05) across the whole phylogeny (4 taxa tree)**

**OrthoDB group id** ^**a**^	**Gene** ^**b**^	**Classification sites** ^**c**^	**Total ratio** ^**d**^	**Likelihood**	**p-value q-value** ^**e**^	**BH-corrected sites** ^**f**^	**Positively selected**
EOG66DJHX-2	dscam	Immunoglobulin	777	186.607	0.00000	0.00000	***4L***, ***6R***, ***8S***, *11D*, ***13G***, ***14D***, ***20Q***, ***22A***, ***24M***, ***26A***, *30T*, ***35A***, ***37T***, ***43E***, ***44P***, *52R*, *54T*, *56I*, ***58T***, ***60P***, *63K*, ***65I***, *66H*
EOG6HHMH6	serpin-23	Scavenger receptor	2066	15.366	0.00046	0.00987	*36S*, *87S*, *90Q*, *92K*, *288P*, *334S*, *397N*, *431S*, *490D*, *761P*, *772R*, *811K*, *815T*, *816Y*, *824S*, *877S*, *1782K*, *1788E*
EOG66DJHQ	aubergine	Small RNA regulatory path-members	787	15.955	0.00034	0.00858	219K, 269K, *287A*, *342S*, 348D, 359G, *397R*, 410E, 415P, *431G*, 436D, *621K*
EOG64B8H5	CLIP-A10	CLIP serine protease	816	29.218	0.00000	0.00003	***1I***, ***3H***, *21V*, *25P*, ***37P***, ***291K***, *333T*, *335T*, ***344S***, ***457S***
EOG6KKWHX	argonaute-2	Small RNA regulatory pathway members	896	20.248	0.00004	0.00150	24W, 27N, 43S, 48Q, 58S, 59N, 81D, 103I, 519F
EOG6J3TZ2	cardinal	Peroxidase	1203	14.276	0.00079	0.01489	*35M*, 46S, 538A, 711A, 742E, 743T, 882D, 931V
EOG6VX0M3	ark	Caspase	1263	17.732	0.00014	0.00423	67G, 386G, 752T, 1028G, 1078T, 1112F
EOG6JQ2CF	LOC100642575 (B. terr)	Scavenger receptor	924	22.212	0.00002	0.00075	75S, 78P, ***111I***, 112P, 647S
EOG6W9GK1-3	rm62-B1	Small RNA regulatory pathway members	431	11.766	0.00279	0.04179	***425A***, *430S*, 431E
EOG634TNR	pellino	Toll pathway	431	12.386	0.00204	0.03407	*1P*, *2S*

^a^Group identifiers are from OrthoDB 6 (http://cegg.unige.ch/orthodb6).

^b^Unless otherwise specified, gene names are taken from the *A. mellifera* or D. melanogaster orthologs.

^c^Total number of codons in the alignment after trimming with Gblocks.

^d^Comparison of model M7 versus M8.

^e^Multiple test correction by the method of Benjamini and Hochberg to control the false discovery rate (only groups where FDR < 0.05 are shown).

^f^Sites are classified as under positive selection if the Bayesian posterior probability > 0.75 (>0.95 in bold italic). Sites where $E\left[\omega \right]-\sqrt{\mathrm{Var}\left(\omega \right)}>1.25$ are italic.

Reference sequence taken from *A. mellifera*.

**Table 5:** **Genes under positive selection (using FDR < 0.05) across the whole phylogeny (5 taxa tree)**

**OrthoDB group id** ^**a**^	**Gene** ^**b**^	**Classification sites** ^**c**^	**Total ratio** ^**d**^	**Likelihood**	**p-value q-value** ^**e**^	**BH-corrected sites** ^**f**^	**Positively selected**
EOG6KKWHX	argonaute-2	Small RNA regulatory pathway members	810	31.839	0.00000	0.00001	*22W*, 25N, 41S, 55S, 56N, 57S, 69L, 73D, 85D, 95I, 329S, 346N, 450F, 692L, 693R
EOG66DJHX-2	dscam	Immunoglobulin	489	44.704	0.00000	0.00000	***2M***, ***4A***, ***13A***, *20R*, *24I*, ***26T***, ***28P***, *31K*, ***33I***, *34H*, *451G*, *452G*
EOG6QRFKP	CLIP-C1B	CLIP serine protease	330	13.650	0.00109	0.03063	14L, 15Q, 66L, 72M, 118A, 132L, 195Q, 313N
EOG6VX0M3	ark	Caspase	1128	17.871	0.00013	0.00619	495S, 629T, 904G, 954T, 988F
EOG64B8H5	CLIP-A10	CLIP serine protease	792	14.259	0.00080	0.02824	1I, 3H, 20V, 318T

^a^Group identifiers are from OrthoDB 6 (http://cegg.unige.ch/orthodb6).

^b^Unless otherwise specified, gene names are taken from the *A. mellifera* or D. melanogaster orthologs.

^c^Total number of codons in the alignment after trimming with Gblocks.

^d^Comparison of model M7 versus M8.

^e^Multiple test correction by the method of Benjamini and Hochberg to control the false discovery rate (only groups where FDR < 0.05 are shown).

^f^Sites are classified as under positive selection if the Bayesian posterior probability > 0.75 (>0.95 in bold italic). Sites where $E\left[\omega \right]-\sqrt{\mathrm{Var}\left(\omega \right)}>1.25$ are Italic.

Reference sequence taken from *A. mellifera*.

**Table 6:** **Genes under positive selection (using FDR < 0.05) on the branch to** ***Apis*** **(5 taxa tree)**

**OrthoDB group id** ^**a**^	**Gene** ^**b**^	**Classification**	**Total sites** ^**c**^	**Likelihood ratio** ^**d**^	**p-value**	**BH-corrected q-value** ^**e**^	**Positively selected sites** ^**f**^
EOG6VX0M3	ark	Caspase	1128	9.974	0.00079	0.02812	412N, 484N, 593S, 862P, 941N, 953L
EOG66Q57J	LOC100642902 (B. terr)	Serine protease inhibitor	1189	9.555	0.00100	0.02812	425D, 452I, 540S, 622S, 721M
EOG634TN0	drosha	Small RNA regulatory pathway members	1290	8.884	0.00144	0.03380	58A, 94N, 155M, 278V
EOG6XWDCW	rm62-C	Small RNA regulatory pathway members	492	10.537	0.00058	0.02812	32S, **130I**, 151S, **269S**
EOG6DV43B	immune deficiency	IMD pathway	249	9.882	0.00083	0.02812	139V, 141S
EOG6RV16R-1	BGRP-1	GNBP	459	9.619	0.00096	0.02812	151L

^a^Group identifiers are from OrthoDB 6 (http://cegg.unige.ch/orthodb6).

^b^Number of codons remaining in the alignment after trimming with Gblocks.

^c^Comparison of Branch-site model A versus a constrained version with *ω*_2_ = 1.

^e^Multiple test correction by the method of Benjamini and Hochberg to control the false discovery rate (only groups where FDR < 0.05 are shown).

^f^Sites are classified as under positive selection if the Bayesian posterior probability > 0.75 (> 0.95 in bold). The reference sequence is from *A. mellifera*.

**Table 7:** **Genes under positive selection (using FDR < 0.05) on the branch to** ***Bombus*** **(5 taxa tree)**

**OrthoDB group id** ^**a**^	**Gene** ^**b**^	**Classification**	**Total sites** ^**c**^	**Likelihood ratio** ^**d**^	**p-value**	**BH-corrected q-value** ^**e**^	**Positively selected sites** ^**f**^
EOG666T1W	domeless	JAK/STAT pathway	1435	9.552	0.00100	0.01951	24L, 102R, 224S, 526A, 770T, 799N, 838V, 942V, 952I, 954A, 959L, 960A, 992Q, 1055R, 1056W, 1312T, 1316D
EOG6VDNFR	dorsal	Relish	353	22.813	0.00000	0.00013	104Q, **177S**, **309R**, 316K, **317I**, **318S**, **332S**, 333Y, **334N**, 336S, 347N, **350R**
EOG66Q57J	LOC100642902 (B. terr)	Serine protease inhibitor	1189	20.354	0.00000	0.00023	165S, 230D, 247P, 419T, 500S, 502D, 590Q, 617S
EOG6BG7B9	snmp1	Scavenger receptor	430	18.183	0.00001	0.00047	**77G**, **217G**, 227K, 346L, 353K, 394N, **395K**
EOG6VX0M3	ark	Caspase	1128	7.406	0.00325	0.04167	156L, 668S, 992R, 1074N, 1079L
EOG6ZPC9T	rm62-F	Small RNA regulatory pathway members	545	11.406	0.00037	0.01032	**120R**, 136G, 169Q, 542N, **543K**
EOG6RV16R-1	BGRP-1	GNBP	459	9.363	0.00111	0.01951	222R, 229E, 370P, 458W
EOG6X0K8Q	myd88	Toll pathway	209	9.782	0.00088	0.01951	45E, 83F, **133P**, 199D
EOG6Z8WBN	catalase	Catalase	181	7.431	0.00321	0.04167	**53A**, 83T, 89S
EOG6931ZS-1	TLR-7	Toll receptor	1299	12.107	0.00025	0.00886	230T, 1190K, 1191D
EOG6KKWHX	argonaute-2	Small RNA regulatory pathway members	810	8.276	0.00201	0.03147	684A

^a^Group identifiers are from OrthoDB 6 (http://cegg.unige.ch/orthodb6).

^b^Number of codons remaining in the alignment after trimming with Gblocks.

^c^Comparison of Branch-site model A versus a constrained version with *ω*_2_ = 1.

^e^Multiple test correction by the method of Benjamini and Hochberg to control the false discovery rate (only groups where FDR < 0.05 are shown).

^f^Sites are classified as under positive selection if the Bayesian posterior probability > 0.75 (> 0.95 in bold). The reference sequence is from *A. mellifera*.

**Table 8:** **Genes under positive selection (using FDR < 0.05) on the branch between** ***Bombus*** **and** ***Apis*** **(4 taxa tree)**

**OrthoDB group id** ^**a**^	**Gene** ^**b**^	**Classification**	**Total sites** ^**c**^	**Likelihood ratio** ^**d**^	**p-value**	**BH-corrected q-value** ^**e**^	**Positively selected sites** ^**f**^
EOG6VDNFR	dorsal	Relish	476	36.202	0.00000	0.00000	121Q, **194S**, **228S**, 318L, **326R**, **330W**, 333K, **334I**, **335S**, **345D**, 348N, **350Q**, **351N**, 353A, **358Y**, **359P**, 363D, **367K**, 368S, 369N, **372D**, **373T**, **375A**, **376K**, **377L**, 380A, 384Q, 386T, **387T**, 390S, **392D**, **394D**, **396C**, **397D**, **398T**, **400T**, **401S**, **403Q**, **404M**, **407F**, 410L, 411S, 415K, **420T**, 422P, **425P**, **433K**, **434Q**, **440V**, 441P, **443E**, **446Q**, **447S**, **448L**, **453N**, 454T, 458S, 462S, **463P**, 465E, **467G**, 468K, **471S**, **472E**, 473K, 474K, 476T
EOG6ZPC96	nimrod-C2	Nimrod	1802	26.848	0.00000	0.00001	37Q, 47Q, 188M, 444H, 458K, 511M, 522V, 535V, 537E, 542Q, 550K, 576C, 582E, 599Y, 612P, 617T, 619V, 626P, 628V, 633R, 643V, 644N, 663R, 669S, 677E, 693S, 1010P
EOG66Q57J	LOC100642902 (B. terr)	Serine protease inhibitor	1327	39.488	0.00000	0.00000	40G, 213S, 287E, 291D, 299V, 334V, 341S, 452S, 503T, 507H, 508S, 509D, 577G, 597D, 631T, 666D, 704K, 708S, 710A, 743S, 758K, 759W, 775Q, 778S, 820K, 856M, 858Q
EOG6BG79T	spindle-E	Small RNA regulatory pathway members	1273	7.774	0.00265	0.03328	17H, 55Q, 157D, 175S, 254N, 391Q, 492G, 749T, 751S, 787I, 832F, 1026P, 1131S, 1237N, 1248T
EOG68SF83	tep23	Thioester-containing protein	1694	10.554	0.00058	0.01087	15T, 39Y, 84S, 204P, 288G, 652A, 683S, 1070S, 1092S, 1466L, 1467S, 1470E, 1482A, 1543L
EOG6QNKCB	spatzle-1B	Spaetzle	169	7.766	0.00266	0.03328	3S, 10C, 14E, 17S, 22A, 36S, 62S, 96A, 116T, 142S
EOG6866VT	tube	Toll pathway	298	8.801	0.00151	0.02258	24S, 30S, 45M, 195L, 267L, **287V**, 295N
EOG6XWDDG-1	serpin-10A	Serine protease inhibitor	385	11.507	0.00035	0.00743	**88S**, 253F, 335S, **341C**, 344P
EOG6W3R35	belle	Small RNA regulatory pathway members	683	14.230	0.00008	0.00303	**134T**, 278I, **602S**, **633Q**, **664S**
EOG6KKWHX	argonaute-2	Small RNA regulatory pathway members	896	12.187	0.00024	0.00665	44S, 49S, 746S
EOG6HHMH6	serpin-23	Scavenger receptor	2066	10.002	0.00078	0.01303	1459K
EOG66DJHX-1	dscam-like protein	Immunoglobulin	1847	12.000	0.00027	0.00665	None

^a^Group identifiers are from OrthoDB 6 (http://cegg.unige.ch/orthodb6).

^b^Number of codons remaining in the alignment after trimming with Gblocks.

^c^Comparison of Branch-site model A versus a constrained version with *ω*_2_ = 1.

^e^Multiple test correction by the method of Benjamini and Hochberg to control the false discovery rate (only groups where FDR < 0.05 are shown).

^f^Sites are classified as under positive selection if the Bayesian posterior probability > 0.75 (> 0.95 in bold). The reference sequence is from *A. mellifera*.

## Discussion

We find that the genomes of both species of bumblebee encode a remarkably similar repertoire of immune-related genes to the honeybee *A.mellifera* and solitary leafcutting bee *M. rotundata* (Figure 2). All the components of the major immune pathways, *Toll, IMD, JAK/STAT, JNK*, and the antiviral machinery are present in both *Bombus* species. Furthermore, the subset of these genes that we surveyed are detectibly expressed and many are induced upon immune activation. Indeed, these immune genes are expressed in a sex-specific fashion as predicted by Bateman’s principle of greater investment into maintenance for the choosier sex, usually females [54]. The sex differences in expression appear to be independent of gene dose since the expression of housekeeping genes was not significantly different between haploid males and gynes.

Overall, the number of immune genes is very consistent among the sequenced bees regardless of their degree of sociality, that is, from solitary (*Megachile*) to primitive (*Bombus*) and higher (*Apis*) eusociality (Figure 2). Primitive eusociality evolved about 87 million years ago in corbiculate bees [55], whereas higher sociality evolved in the Apini and Meliponi/Bombini with sociality being presumably secondarily lost in the Euglossini [55]. According to our results, the solitary bee *M. rotundata*, which split from the Apidae some 105 million years ago, has a comparable number of immune genes to honeybees and both *Bombus* species. These results suggest that the immune repertoire of *A. mellifera*, which was described as depauperate relative to dipterans, is probably a characteristic of bees more generally and predates the evolution of sociality and certainly existed before advanced eusociality in bees, and perhaps even as far back as before the split with ants [20]. Therefore, the relatively limited immune repertoire of honeybees does not seem to be the result of the transition to sociality and the associated behavioral adaptations for social immunity as suspected before [16]. An intriguing but purely speculative thought is that, rather than sociality reducing the need for immune genes, reduced immune complexity may have facilitated (for example, by way of easing the self/foreign distinction) or empowered (by way of allowing for social defenses) the evolution of social groups in the first place.

Both *Bombus* species have a small expansion of serpins (Figure 3). These serpins appear similar to the silkworm moth *B. mori*’s *antitrypsin*, which is involved in prophenoloxidase (PPO) regulation and is upregulated upon fungal infection [56]. We confirmed that these serpins are expressed in *B. terrestris* when challenged and are thus likely functional. The honeybee homolog seems to have a mutation within the binding region PS00284, which does not conform to the consensus pattern of this active site. It is unclear whether this gene in honeybees is a functional serpin. We also find a caspase that is similar to *Decay* in *D. melanogaster* (Figure 4), which has not been found in either *A. mellifera* or *Nasonia vitripennis*.

Despite having simpler colony organization and shorter colony lifespan, both bumblebee species nevertheless appear largely like honeybees in their immune-gene characteristics. Indeed, they also appear similar to the solitary leaf-cutting bee *M. rotundata.* While the complement of canonical immune genes may be consistent, it is important to recognize that our understanding of immunity is largely based on the known repertoire of non-social insects, and in particular the fruit fly *D. melanogaster.* As such, we are limited in being able to identify only known immune genes that have been functionally characterized in model systems. Bees may have further unexplored immune genes, novel defenses, and social behaviors that aid disease control and are unavailable to solitary species [21]. These adaptations are also genetically controlled, but the genes behind these traits are less well defined than the canonical immune response genes. Thus, while the Apoidea may appear to have consistent immune genomic profiles at the level of genes shared, they may differ considerably in the genetic underpinning of other key aspects of disease control in a social context, such as grooming, nest hygiene, and other behaviors. As a class, immune genes are rapidly evolving [57-60]. Here we explored which, among these immune genes, show particularly rapid evolution, or differences in selection among the different clades investigated. We found that some genes are under stronger selection in *Bombus* compared with *Apis* (genes below the diagonal in Figure 9), and a number of genes are under stronger positive selection in the social clade (upper diagonal in Figure 9) than in *M. rotundata*. While it is likely that clades with ω > 1 are under positive selection, these results should also be interpreted cautiously because without population data it is not possible to distinguish positive selection from relaxed constraints on selection [61]. Interestingly, we found a strong signature of selection on *dscam*, a gene primarily important for neuronal self-avoidance, but that is increasingly of interest in host-parasite interactions because alternative splicing of this gene can theoretically produce over 150,000 isoforms in *D. melanogaster* [62]. As such, *dscam* is hypothesized to be important for host-parasite specificity in susceptibility, and for specific immune memory [63]. The region under selection in *dscam* is limited to the beginning of the aligned protein (Figure 7A). This region corresponds to the fifth immunoglobulin I-set domain (sixth immunoglobulin domain overall). All of the previous immunoglobulin domains (1 to 5) were trimmed because they were not present in the *A. mellifera* gene. This gene appears to be under selection at least in the fifth immunoglobulin I-set domain but may also be variable in earlier domains. A previous study that examined the sequence of alternatively splicing exon cassettes did not detect selection in the crustacean *Daphnia magna* and several *Drosophila* species, at immunoglobulin (Ig) 2, 3 and 7 [64]. Our domain, however, likely corresponds to Ig4 or 5 in [64] and thus was not directly tested in their analysis. Nevertheless, our analysis is suggestive of differences in selective pressures among bee species. Among the other genes that show evidence of selection are a number of antiviral genes, including *argonaute 2*, *aubergine* (Figure 8A, B), and *dicer 2*, all of which have been found in other systems to be under selection [60,65]. We also detect evidence of selection on two AMPs, *abaecin* and *defensin* (Additional files 8, 10, and 11), both of which appear to be under stronger selection in the *Apis* clade (Figure 9). Our results corroborate those of Erler *et al.* [66], who also found positive selection on AMPs across several European bumblebee species. Interestingly, we find that *dorsal* appears to be under different selection in bumblebees than in honeybees, where Harpur and Zayed [61] found that *dorsal* was under purifying selection. We also find that all but one of the sites under selection in *dorsal* are outside of the relish domain (Figure 7C). Population level studies of the genes that appear to be evolving under different pressures in honeybees and bumblebees, and in the social and solitary clades will be instrumental in determining which of these genes are evolving under positive, relaxed or balancing selection [61].

## Conclusions

Social insects have a suite of adaptations that have been hypothesized to reduce the pressure on immune system evolution, to the point of widespread gene losses, or inversely failing to produce or maintain duplicates. However, we find no evidence of great variation in immune gene complement, or in terms of the total number of immune-related genes across a gradient of sociality (highly social *Apis* > primitively social *Bombus* > solitary *M. rotundata*). Instead, we find a more nuanced pattern of immune system evolution, with variation in signatures of selection among these taxa. The different selective pressures that drive the evolution of these immune genes may in turn reflect the different parasite pressures and life history characteristics of different bee species. The depauperate immune repertoire of honeybees relative to model species thus appears to be ancestral to the evolution of bee sociality and not a consequence of sociality.

## Materials and methods

### Survey for immunological repertoire and annotation

The genomes of haploid males from a single colony of *B. terrestris* and of *B. impatiens* were sequenced by the Bumblebee Genome Consortium and the details of the sequencing, assembly, and automated annotation can be found in [67]. Using OrthoDB [68,69] orthologous groups, we identified orthologs from the two bumblebees, as well as from *Apis florea*, *M. rotunda*, and *N. vitripennis*, *Tribolium castaneum*, of previously characterized immune genes from *D. melanogaster*, *A. gambiae*, and *A. mellifera* that comprise 27 immune-related gene families or pathways. To complement the orthology searches, we searched for homologs of known immune proteins from the two bumblebees using blastp [70,71] against the official gene sets (NCBI RefSeqs). To confirm the absence of any proteins that appeared to be missing, we searched the genome assemblies and Short Reads Archive using tblastn.

### Immunological expression

To confirm the relevance of these genes to immune activation and the validity of novel genes revealed in our annotation we challenged 2- to 3-week-old unmated male and gyne (that is, daughter queen) *B. terrestris* by injecting them with 2 μl of a suspension of either heat-killed *E. coli* (Gram-negative) or *Arthrobacter globiformis* (Gram-positive) at a concentration of 10^8^ cfu/ml, or with sterile Ringer solution under the tergites of the abdomen, or as naïve controls handled them in the same way without any injection. We used 12 replicates for each treatment/caste combination (total N = 96). These experimental bees were the granddaughters and grandsons of queens collected in northern Switzerland in spring 2012 that had established colonies in the lab. We confirmed that these colonies were free of common parasites such as *Crithidia bombi* and *Nosema bombi* through visual inspection. All experimental bees were immobilized on ice for 30 minutes before treatment, including the naïve controls. After treatment we housed the bees singly with *ad libitum* pollen and 50% (w/w) sugar water. Eight hours after treatment we snap froze the bees in liquid nitrogen. We homogenized the abdomens before extraction with 0.5 g Zirkonium beads at 0°C to −4°C using an Omni Bead Ruptor 24 Homogenizer (OMNI International, Kennesaw, GA, USA). We then extracted total RNA using Qiagen RNeasy Plus Mini extraction kits (Qiagen, Hilden, Germany) according to the manufacturer's instructions. We confirmed RNA integrity of every sample with 2100 Bioanalyzer (Agilent Technologies, Santa Clara, CA, USA) with the RNA 6000 Nano Kits. We transcribed the RNA to cDNA using Quantitect reverse transcription kits (Qiagen) including controls without reverse transcriptase (no-RT controls) to test for genomic contamination. All samples were checked using quantitative PCR for our housekeeping genes to ensure that the no-RT controls amplified at least 10 cycles later, and thus contain less than 0.1% of the transcripts found in the RT samples.

Based on the full genomic sequences, we selected 27 candidate genes to represent various components of the immune response of insects, including the Toll, JAK/STAT, IMD and JNK pathways; recognition genes, melanization responses, various effectors and antiviral genes. We explored the expression of these genes upon immune stimulation relative to the geometric mean of three housekeeping genes (*pla2*, *ak*, *ef1a*). The full list of genes, their accession numbers and primers can be found in Table 1. All primers were designed using QuantPrime [72], based on the GenBank sequences (Table 1), except those for *relish*, which were published in [73]. The primers for *kayak* were designed based on a manually annotated gene given the temporary identifier (Bter:08277927). All primers were tested and have minimal dimer and high amplification efficiency (1.9 to 2.1).

We measured expression on a Fluidigm 96.96 Dynamic array on the BioMark system (Biomark Inc., Pueblo, CO, USA) using EvaGreen as a reporter according to the manufacturer’s protocol (Advanced Development Protocol 14; PN 100–1208 B). We eventually measured expression of 95 samples (12 replicates for each treatment in males and in queens with one naïve queen randomly dropped to make room for the negative control). We ran the samples with three technical replicates and used the average cycle threshold (Ct) of these technical replicates for further analysis.

We standardized expression of all genes of interest relative to the geometric mean of our three housekeeping genes (yielding deltaCt (dCt) values; all dCT values first transformed with Yeo-Johnson power transformations to improve normality and homoscedasticity, 'car' package in R) after confirming that the composite housekeeping value did not vary with sex (*F*_1,87_ = 0.09, *P* = 0.77), treatment (*F*_3,87_ = 0.29, *P* = 0.83), or their interaction (*F*_3,87_ = 0.70, *P* = 0.56) by ANOVA. We analyzed these dCt values using MANOVA with sex (gyne and male) and treatment (naïve, injected with Ringer’s solution, heat-killed *E. coli,* or heat-killed *A. globiformis*) as fixed, fully crossed factors (base package in R). We used MANOVA for these analyses, since the expression of any of these genes is not independent of the expression of other genes and because MANOVA accounts for multiple testing and is thus robust to type I error. When MANOVA effects were significant, we subsequently explored the univariate individual gene effects.

### Building gene family phylogenies

We retrieved protein sequences of selected gene families from OrthoDB [68,69] and aligned them using ClustalW [74] and adjusted the alignments manually or with Gblocks [75] before tree-building using MrBayes [76] with the mixed model. We ran MrBayes for as long as was necessary (typically for 20,000 to 400,000 generations) to reduce the average deviation of split frequencies below 0.01 or until the split frequency approached 0.01 but did not improve further. We discarded the initial 25% of the trees as a burn-in.

### Testing for signatures of selection

We extracted orthologous groups of immune-related genes from OrthoDB6 [68,69]. From the 130 orthologous groups with sequences from *B. terrestris*, *B. impatiens*, *A. mellifera* and *A. florea* we extracted 148 multiple sequence alignments containing exactly one sequence from each species. We use these 148 alignments for comparisons between the *Bombus* and *Apis* clades. From the 122 orthologous groups that contain *M. rotundata* sequences we further extracted 139 alignments that also contain a *M. rotundata* ortholog, which we use as an outgroup to compare social with solitary (non-social) bees. In six of the alignments (*abaecin*, *basket*, *cactus*, *defensin*, *kayak* and *tak1*) one or more orthologs were not present in OrthoDB6 and had to be retrieved from an alternative source (that is, NCBI). Protein sequences were aligned independently for the four-taxa (*Bombus* and *Apis*) or five-taxa trees (with *Megachile*) with ProGraphMSA [77] and trimmed using Gblocks with the stringent settings as described in [75]. Where orthologous groups contained multiple sequences for some species the most closely related sets of sequences were aligned. In the 12 orthologous groups that contained more than one sequence for each species we extracted the maximum number of alignments, such that each alignment contains only one sequence from each species. We retrieved cDNA sequences for the alignments from the official gene sets (*A. mellifera* v.4.5, *A. florea* v.1.0, *B. impatiens* v.2.0, *B. terrestris* v.1.0, *M. rotundata* v.1.0) using a custom written Python script.

We used likelihood-based codon models implemented in the PAML package [78] to analyze differences in the rate of evolution and to test for signals of selection. We tested hypotheses by using likelihood ratio tests to select the best fitting model among pairs of nested models that differ only in their representation of ω, the ratio of non-synonymous to synonymous substitutions (ω = dN/dS). We make the assumption that ω > 1 indicates positive selection, while ω < 1 and ω = 1 indicates negative and neutral selection.

The average rate of evolution was determined using the M0 [79] model, which assumes a constant ω across all sites and branches. The average ω is not a good indicator for the presence of positive selection, since functional and structural constraints ensure that most sites in functional genes are conserved [80]. Hence, we used the M7 and M8 models to test for the presence of positively selected sites. [79]. Both models allow ω to vary from site-to-site according to a Beta distribution, but the M8 model additionally allows some sites to evolve with ω > 1, to account for sites under positive selection.

In order to detect episodes of positive selection on the connecting branches between clades we used the branch-site model [81,82]. Some branches are assigned *a priori* to the foreground, where some sites are allowed to evolve with ω > 1, while all sites on background branches are constrained to ω ≤ 1. The branch-site model is compared to a null model where there is no difference between foreground and background branches. We used Clade model D [83] to test for more general differences between clades. This model allows ω to differ between clades in some sites. It is compared to a null model where there are no differences in ω between clades.

To ensure that the PAML optimization did not get stuck in local optima we used six different initial estimates for ω in all analyses and initialized branch lengths to values calculated with PhyML [84]. We corrected for multiple testing by controlling the false discovery rate using the method of Benjamini and Hochberg [85]. To calculate the posterior probabilities of sites being under positive selection in the M8 and Branch-site models we used the Bayes Empirical Bayes (BEB) approach implemented in PAML [86].

We repeated the analyses using Probcons [87] for aligning sequences. However, we only report the results from alignments produced by ProGraphMSA, as these alignments give more conservative estimates and hence a smaller chance of falsely reporting positive selection. Similarly, we do not report results from using Gblocks with the relaxed settings, as described in [75], or no trimming at all. These results are available from the authors.

### Data

Sequence data can be found on NCBI (*B. impatiens* BIMP_2.0 RefSeq Assembly GCF_000188095.1, *B. terrestris* Bter_1.0 GCF_000214255.1, *A. mellifera* Amel_4.5 GCF_000002195.4, *A. florea* Aflo_1.0 GCF_000184785.1, *M. rotundata* MROT_1.0 GCF_000220905.1). Alignments used in this manuscript can be found in Additional files 12.

## Additional files

Additional file 1:
**Statistics for the global ω ratio obtained by the M0 model (4 taxa tree).**
Additional file 2:
**Statistics for the global ω ratio obtained by the M0 model (5 taxa tree).**
Additional file 3:
**The 30 fastest evolving genes as determined by the M0 model (4 taxa tree).**
Additional file 4:
**The 30 slowest evolving genes as determined by the M0 model (4 taxa tree).**
Additional file 5:
**The 30 fastest evolving genes as determined by the M0 model (5 taxa tree).**
Additional file 6:
**The 30 slowest evolving genes as determined by the M0 model (5 taxa tree).**
Additional file 7:
**Genes under positive selection (using FDR < 0.05) on the branch to Megachile (5 taxa tree).**
Additional file 8:
**Genes that tested significant for evolving under differenct selective pressures (FDR < 0.05) between Bombus and Apis according to Clade model D (4 taxa tree).**
Additional file 9:
**Genes that tested significant for evolving under different selective pressures (FDR < 0.05) between the social and solitary clades according to Clade model D (5 taxa tree).**
Additional file 10:
**Summary of models on the 4 taxa tree.**
Additional file 11:
**Summary of models on the 5 taxa tree.**
Additional file 12:
**Amino acid alignments used for PAML analyses.**
Additional file 13:
**Statistical results for MANOVA and subsequent ANOVA of gene expression data.**

## Abbreviations

AMP: antimicrobial peptide
NCBI: National Center for Biotechnology Information
PCR: polymerase chain reaction
RT: reverse transcriptase
