# Supplementary material for: A depauperate immune repertoire precedes evolution of sociality in bees
Source: Genome Biol. 2015 Apr 24;16(1):83. doi: 10.1186/s13059-015-0628-y (PMC4408586; doi:10.1186/s13059-015-0628-y)
Supplement: Additional file 1: — Statistics for the global ω ratio obtained by the M0 model (4 taxa tree). [file 13059_2015_628_MOESM1_ESM.pdf]

Statistics for the global ω ratio obtained by the M0 model (4 taxa tree).

	Global ω	Tree length (dN)	Tree length (dS)
Mean	0.11557	0.17356	1.66915
Median	0.09789	0.08910	0.91565
Variance	0.00750	0.17117	17.27791
Standard deviation	0.08661	0.41373	4.15667
Standard error	0.00707	0.03378	0.33939
