# Supplementary material for: A depauperate immune repertoire precedes evolution of sociality in bees
Source: Genome Biol. 2015 Apr 24;16(1):83. doi: 10.1186/s13059-015-0628-y (PMC4408586; doi:10.1186/s13059-015-0628-y)
Supplement: Additional file 2: — Statistics for the global ω ratio obtained by the M0 model (5 taxa tree). [file 13059_2015_628_MOESM2_ESM.pdf]

Statistics for the global ω ratio obtained by the M0 model (5 taxa tree).

	Global ω	Tree length (dN)	Tree length (dS)
Mean	0.09567	0.28551	3.34022
Median	0.07891	0.16430	1.87740
Variance	0.00473	0.16945	25.99466
Standard deviation	0.06881	0.41165	5.09850
Standard error	0.00579	0.03467	0.42937
