# Supplementary material for: A depauperate immune repertoire precedes evolution of sociality in bees
Source: Genome Biol. 2015 Apr 24;16(1):83. doi: 10.1186/s13059-015-0628-y (PMC4408586; doi:10.1186/s13059-015-0628-y)
Supplement: Additional file 3: — The 30 fastest evolving genes as determined by the M0 model (4 taxa tree). [file 13059_2015_628_MOESM3_ESM.pdf]

The 30 fastest evolving genes on the 4 taxa tree as determined by the M0 model

OrthoDB group[a]	Gene[b]	Classification	Global ω[c]	Tree Length[d]
EOG6TX97R	CLIP–D1–like protein	CLIP serine protease	0.36655	1.2491
EOG6JSXPT	abaecin	AMP	0.33584	0.9080
EOG6QRFKP	CLIP–C1B	CLIP serine protease	0.32940	1.0886
EOG6HDR8C	TLR–1	Toll receptor	0.31941	1.3837
EOG6RJDH9	scarface	CLIP serine protease	0.30015	1.0860
EOG66Q57J	LOC100642902 (B. terr)	Serine protease inhibitor	0.29770	1.0302
EOG6QNKCB	spatzle–1B	Spaetzle	0.29567	3.1566
EOG680GDM	eiger	c–Jun N–terminal kinases	0.27614	1.0018
EOG6RV16R–1	BGRP–1	GNBP	0.27490	1.0508
EOG6RBP1C	persephone	CLIP serine protease	0.27289	1.1464
EOG6FJ6RD	CLIP–B13	CLIP serine protease	0.26853	0.9685
EOG6NCJV4	snake	CLIP serine protease	0.26193	0.9349
EOG6WDBSW	serpin–28D	Serine protease inhibitor	0.25778	1.2144
EOG66DJHQ	aubergine	Small RNA regulatory pathway members	0.25716	1.3249
EOG69CNQ4–2	IAP–1B	IAP repeat	0.24138	3.1745
EOG6TX97G	CLIP–D1	CLIP serine protease	0.22975	1.2046
EOG6ZW3V0	fadd	IMD pathway	0.22926	1.0050
EOG6RJDHD	r2d2	Small RNA regulatory pathway members	0.22813	1.1397
EOG6RV16R–2	BGRP–2	GNBP	0.22745	1.0129
EOG6866VT	tube	Toll pathway	0.22611	1.0925
EOG6KKWHX	argonaute–2	Small RNA regulatory pathway members	0.22524	1.2634
EOG6VX0NG–3	PGRP–S1	PRGP	0.22490	0.9712
EOG6M37R0	serpin–27A	Serine protease inhibitor	0.21958	1.0711
EOG6HHMH6	serpin–23	Scavenger receptor	0.21578	0.8905
EOG6XWDDG–3	serpin–10B	Serine protease inhibitor	0.21295	1.2529
EOG61NS2P	CLIP–A5	CLIP serine protease	0.21220	1.0629
EOG6RBP1B	cactus	Toll pathway	0.21105	1.4408
EOG6TDZ3C	defensin	AMP	0.20810	1.0139
EOG6DV43B	immune deficiency	IMD pathway	0.20362	0.9836
EOG6VX0NG–2	PGRP–S2	PRGP	0.19318	0.9017

[a] Group identifiers are from OrthoDB 6 (http://cegg.unige.ch/orthodb6).
[b] Unless otherwise specified, gene names are taken from the *A. mellifera* or *D. melanogaster* orthologs.
[c] Maximum likelihod estimate across all sites and branches.
[d] Tree length in synonymous substitutions per synonymous sites.
