# Supplementary material for: A depauperate immune repertoire precedes evolution of sociality in bees
Source: Genome Biol. 2015 Apr 24;16(1):83. doi: 10.1186/s13059-015-0628-y (PMC4408586; doi:10.1186/s13059-015-0628-y)
Supplement: Additional file 4: — The 30 slowest evolving genes as determined by the M0 model (4 taxa tree). [file 13059_2015_628_MOESM4_ESM.pdf]

The 30 slowest evolving genes on the 4 taxa tree as determined by the M0 model

OrthoDB group[a]	Gene[b]	Classification	Global ω[c]	Tree Length[d]
EOG60001Z	atg8	Autophagy	0.00010	0.4288
EOG6XPNXB	basket	c–Jun N–terminal kinases	0.00435	0.5255
EOG6VX0M4	rm62–I	Small RNA regulatory pathway members	0.00691	1.2326
EOG6GHX4B	CLIP–D10	CLIP serine protease	0.00845	1.0736
EOG6WH726	atg6	Autophagy	0.01014	0.6817
EOG6NVX17	argonaute–1	Small RNA regulatory pathway members	0.01077	0.4826
EOG6PC86T	tor	Autophagy	0.01111	0.7607
EOG6DR7V8	atg18	Autophagy	0.01356	0.3759
EOG641NSQ	stat	JAK/STAT pathway	0.01621	0.8793
EOG634TNR	pellino	Toll pathway	0.01657	0.6012
EOG6J9KDP	Duox	Peroxidase	0.01738	0.7475
EOG6W9GK1–2	rm62–H	Small RNA regulatory pathway members	0.01879	12.8089
EOG64B8HP	kayak	c–Jun N–terminal kinases	0.01900	0.5607
EOG6K3JCB	jra	c–Jun N–terminal kinases	0.02014	1.1542
EOG6931ZS–4	TLR–8	Toll receptor	0.02026	1.4883
EOG6GXD37	pasha	Small RNA regulatory pathway members	0.02037	0.9185
EOG69W0XF	ran	Small RNA regulatory pathway members	0.02071	0.8633
EOG6P8D0V	atg5	Autophagy	0.02194	0.6426
EOG6KKWJ6	fmr1	Small RNA regulatory pathway members	0.02329	0.3478
EOG6612K2	rm62–A	Small RNA regulatory pathway members	0.02393	0.7787
EOG634TMW	ranbp–21	Small RNA regulatory pathway members	0.02405	0.7290
EOG66DJHX–1	dscam–like protein	Immunoglobulin	0.02561	23.4644
EOG6W9GK1–4	rm62–J1	Small RNA regulatory pathway members	0.02738	11.6353
EOG6W6MC0	Clect–GA3	C–type lectin	0.02812	0.8423
EOG6931ZS–2	TLR–10	Toll receptor	0.02863	1.6222
EOG66WWRH–1	Clect–GA1	C–type lectin	0.02995	1.0848
EOG66T1GW	caspar	IMD pathway	0.03108	0.6715
EOG6931ZS–1	TLR–7	Toll receptor	0.03131	0.9871
EOG6931ZS–3	TLR–6	Toll receptor	0.03138	0.7313
EOG6TTF0B	spatzle–6	Spaetzle	0.03255	0.8090

[a] Group identifiers are from OrthoDB 6 (http://cegg.unige.ch/orthodb6).
[b] Unless otherwise specified, gene names are taken from the *A. mellifera* or *D. melanogaster* orthologs.
[c] Maximum likelihod estimate across all sites and branches.
[d] Tree length in synonymous substitutions per synonymous sites.
