# Supplementary material for: A depauperate immune repertoire precedes evolution of sociality in bees
Source: Genome Biol. 2015 Apr 24;16(1):83. doi: 10.1186/s13059-015-0628-y (PMC4408586; doi:10.1186/s13059-015-0628-y)
Supplement: Additional file 5: — The 30 fastest evolving genes as determined by the M0 model (5 taxa tree). [file 13059_2015_628_MOESM5_ESM.pdf]

The 30 fastest evolving genes on the 5 taxa tree as determined by the M0 model

OrthoDB group[a]	Gene[b]	Classification	Global ω[c]	Tree Length[d]
EOG6TX97R	CLIP–D1–like protein	CLIP serine protease	0.30094	2.2282
EOG6HDR8C	TLR–1	Toll receptor	0.25801	2.7466
EOG6RJDH9	scarface	CLIP serine protease	0.25097	1.9614
EOG6RV16R–1	BGRP–1	GNBP	0.24922	2.1086
EOG66Q57J	LOC100642902 (B. terr)	Serine protease inhibitor	0.24741	2.3151
EOG6RBP1C	persephone	CLIP serine protease	0.24712	2.2103
EOG6QRFKP	CLIP–C1B	CLIP serine protease	0.24449	2.3723
EOG6NCJV4	snake	CLIP serine protease	0.23339	1.6048
EOG6FJ6RD	CLIP–B13	CLIP serine protease	0.22864	1.9741
EOG64QRG8	tak1	c–Jun N–terminal kinases	0.22180	14.7534
EOG6KKWHX	argonaute–2	Small RNA regulatory pathway members	0.22158	2.6678
EOG6XWDDG–3	serpin–10B	Serine protease inhibitor	0.20280	3.0118
EOG6ZW3V0	fadd	IMD pathway	0.20183	2.3310
EOG6RJDHD	r2d2	Small RNA regulatory pathway members	0.20087	2.1837
EOG6HHMH6	serpin–23	Scavenger receptor	0.19767	1.7375
EOG680GDM	eiger	c–Jun N–terminal kinases	0.19204	2.7384
EOG6WDBSW	serpin–28D	Serine protease inhibitor	0.19085	2.1584
EOG6RV16R–2	BGRP–2	GNBP	0.18544	2.1662
EOG6N8PNJ	wengen	c–Jun N–terminal kinases	0.18343	0.8607
EOG64J10C	ird5	IMD pathway	0.18295	1.6195
EOG66DJHQ	aubergine	Small RNA regulatory pathway members	0.17942	2.4938
EOG6TDZ3C	defensin	AMP	0.17525	1.7331
EOG6TX97G	CLIP–D1	CLIP serine protease	0.17348	2.7346
EOG6RBP1B	cactus	Toll pathway	0.17196	2.2317
EOG6W0VV6	SCR–B5	Scavenger receptor	0.16861	1.7944
EOG6FBG9K	npc2a	MD–2–related lipid recognition	0.16468	1.8724
EOG6XWDDG–1	serpin–10A	Serine protease inhibitor	0.16206	7.7541
EOG6NVX30	npc2b	MD–2–related lipid recognition	0.16092	1.2163
EOG62BVQX	caspase–L1	Caspase	0.15962	2.5414
EOG6X3FGJ	SCR–B6	Scavenger receptor	0.15942	2.0908

[a] Group identifiers are from OrthoDB 6 (http://cegg.unige.ch/orthodb6).
[b] Unless otherwise specified, gene names are taken from the *A. mellifera* or *D. melanogaster* orthologs.
[c] Maximum likelihod estimate across all sites and branches.
[d] Tree length in synonymous substitutions per synonymous sites.
