# Supplementary material for: A depauperate immune repertoire precedes evolution of sociality in bees
Source: Genome Biol. 2015 Apr 24;16(1):83. doi: 10.1186/s13059-015-0628-y (PMC4408586; doi:10.1186/s13059-015-0628-y)
Supplement: Additional file 6: — The 30 slowest evolving genes as determined by the M0 model (5 taxa tree). [file 13059_2015_628_MOESM6_ESM.pdf]

The 30 slowest evolving genes on the 5 taxa tree as determined by the M0 model

OrthoDB group[a]	Gene[b]	Classification	Global ω[c]	Tree Length[d]
EOG6XPNXB	basket	c–Jun N–terminal kinases	0.00469	0.9624
EOG6VX0M4	rm62–I	Small RNA regulatory pathway members	0.00638	1.8385
EOG69W0XF	ran	Small RNA regulatory pathway members	0.00899	2.0558
EOG6PC86T	tor	Autophagy	0.00972	1.8615
EOG6GHX4B	CLIP–D10	CLIP serine protease	0.01051	1.5225
EOG634TNR	pellino	Toll pathway	0.01095	1.3130
EOG6WH726	atg6	Autophagy	0.01267	1.6741
EOG6J9KDP	Duox	Peroxidase	0.01440	1.7390
EOG6W9GK1–2	rm62–H	Small RNA regulatory pathway members	0.01533	26.8464
EOG641NSQ	stat	JAK/STAT pathway	0.01591	1.9244
EOG6K3JCB	jra	c–Jun N–terminal kinases	0.01599	1.9814
EOG6931ZS–4	TLR–8	Toll receptor	0.01807	2.0594
EOG6CRJF5	masquerade	CLIP serine protease	0.01809	2.4697
EOG6NVX17	argonaute–1	Small RNA regulatory pathway members	0.01837	1.1468
EOG6612K2	rm62–A	Small RNA regulatory pathway members	0.01941	1.5797
EOG634TMW	ranbp–21	Small RNA regulatory pathway members	0.02043	1.4494
EOG6W9GK1–3	rm62–B1	Small RNA regulatory pathway members	0.02174	12.2854
EOG60001Z	atg8	Autophagy	0.02379	0.9714
EOG6X3FG7	tudor–SN	Small RNA regulatory pathway members	0.02586	1.6953
EOG6P8D0V	atg5	Autophagy	0.02650	1.7760
EOG6KKWJ6	fmr1	Small RNA regulatory pathway members	0.02706	0.7046
EOG66WWRH–1	Clect–GA1	C–type lectin	0.02727	15.4043
EOG66DJHX–1	dscam–like protein	Immunoglobulin	0.02775	39.7524
EOG6W9GK1–1	rm62–J	Small RNA regulatory pathway members	0.02782	14.5619
EOG6931ZS–1	TLR–7	Toll receptor	0.02839	1.6509
EOG64B8HP	kayak	c–Jun N–terminal kinases	0.02866	1.1186
EOG6K6DKF	cactin	Toll pathway	0.03088	2.3047
EOG6KD521	chorion peroxidase	Peroxidase	0.03149	16.4281
EOG6DR7V8	atg18	Autophagy	0.03276	1.0310
EOG6TTF0B	spatzle–6	Spaetzle	0.03401	1.7632

[a] Group identifiers are from OrthoDB 6 (http://cegg.unige.ch/orthodb6).
[b] Unless otherwise specified, gene names are taken from the *A. mellifera* or *D. melanogaster* orthologs.
[c] Maximum likelihod estimate across all sites and branches.
[d] Tree length in synonymous substitutions per synonymous sites.
