# Supplementary material for: A depauperate immune repertoire precedes evolution of sociality in bees
Source: Genome Biol. 2015 Apr 24;16(1):83. doi: 10.1186/s13059-015-0628-y (PMC4408586; doi:10.1186/s13059-015-0628-y)
Supplement: Additional file 7: — Genes under positive selection (using FDR < 0.05) on the branch to Megachile (5 taxa tree). [file 13059_2015_628_MOESM7_ESM.pdf]

Genes under positive selection (using FDR < 0.05) on the branch to *Megachile* (5 taxa tree)

OrthoDB Group[a]	Gene[b]	Classification	Sites[c]	*p*–value[d]	*q*–value[e]	Positively selected sites[f]
EOG6KD521	chorion peroxidase	Peroxidase	352	0.00003	0.00065	21A, **60S**, 125L, 127L, 128T, **133L**, **135A**, **137E**, 138H, 140R, 141I, 146I, 148I, **152W**, **153D**, **154D**, **155E**, **158Q**, **160A**, **161R**, **163I**, 164V, 166A, 169Q, **170H**, **171I**, 176F, **178P**, **179I**, 183S, **185I**, **186D**, 187A, **188F**, **189A**, 191A, 192A, **195F**, **196G**, 199L, 200L, **203A**, **204V**, 206R, **207W**, **209K**, **210A**, **212K**, **213F**, 215A, 216S, **217K**, 218R, **219L**, **220S**, 222L, 223I, **225R**, **227F**, **228D**, 229L, **230Y**, 231R, **232A**, **233G**, **234V**, **236D**, **238Y**, 240M, **241G**, **243M**, **244N**, 247A, **248Q**, 249A, 250M, **252D**, **253S**, **255T**, 256Q, **259T**, 260N, **263F**, **264K**, **267G**, **269K**, **270F**, **271G**, 272L, 274L, 275V, **276S**, **279M**, 281R, **282G**, **286P**, 290I, **293Y**, **296I**, 297F, 298E, 299H, 305L, **306W**, 308P, **310P**, 314R, **318A**, **319R**, **320V**, **323D**, **324N**, 327L, **328I**, **329D**, **330T**, **336M**, 339P, **341H**, **349C**, **350R**, **351S**, 352G
EOG6XWDDG–1	serpin–10A	Serine protease inhibitor	375	0.00014	0.00183	2S, 3A, **6N**, 8E, 9N, 14L, 15S, 19N, 23R, 25L, 26Y, 31S, **40S**, 42L, **46M**, 49S, 50H, 51L, 55A, **56E**, **57S**, **60L**, **62E**, 64T, 70Y, 74L, 78E, **81S**, 83I, **86L**, 92V, 94L, 95Y, 96I, 99A, 101Y, 103Q, 105G, **106F**, 109L, **111E**, 112F, 116Q, **117S**, 119I, **120S**, 121K, 125K, 128V, 133K, 136A, 141K, 145K, **152S**, 156N, 172T, 176I, 179I, 180R, 185R, 192N, **194T**, **195K**, 196L, 199T, 201Y, 202S, **204S**, 211M, 212P, 217K, 218F, 221I, 226P, 231I, 235N, 236Q, 239G, 243L, 244Q, 245T, 246N, **247F**, 251M, 253A, 256T, 257R, 259Y, 260N, 264L, **265Y**, 269F, **273F**, 274T, **276D**, 286L, 288T, 290F, 294A, 297N, 298R, 300S, 305M, 310L, **312K**, 314I, 323E, **330R**, **332R**, 338E, 341E, 349M, 351I, 353E, 358N, 359I, 360P, 366V, 368V, 375L
EOG634TMX	dicer–2	Small RNA regulatory pathway members	1269	0.00090	0.00793	7E, 22C, 23K, 59G, 86S, 95G, 133N, 134R, 161N, 225D, 232N, 233N, 244L, 248E, **256D**, 310H, 410N, 454S, 458Q, 461C, 531S, 555G, 617W, 632Y, 660T, 707E, 756K, 765N, 766N, 913S, 938A, 1011R, 1039F, 1052Y, 1176N, 1181V, 1182V, 1220K
EOG66Q57J	LOC100642902 (B. terr)	Serine protease inhibitor	1189	0.00000	0.00000	143V, 154S, 215Y, 239K, 251E, 261E, 275D, 292P, 322Y, 334K, 370T, 382E, 410I, 443H, 469P, 496S, 564N, 571S, 593D, 596Y, 604S, 619V, 631D, 668D, 675K, 676T, 686S, 687M, 716E, 735S, 742S, 745T, 748N, 796S
EOG6FN2ZK	hopscotch	JAK/STAT pathway	600	0.00000	0.00000	6L, 20L, 68M, **354D**, **356C**, **360G**, 361K, **365P**, 366P, 488F, 489E, 507S, 532T, 536Y, 586R, 587L, 588Q, 591Q, **594G**, **595S**, **597E**, **598L**, **599L**
EOG6Q2BWQ	IAP–2	IAP repeat	503	0.00000	0.00011	114K, 157E, 198N, **202N**, 204S, **440E**, 441T, 443L, 444E, **449K**, **460M**, 479Y, **483S**, 484L, 486Y, **493E**, 495K, 496A
EOG608KPT	LOC100642932 (B. terr)	Scavenger receptor	1646	0.00000	0.00001	74N, 249N, 254P, 332K, 347S, 378Y, 381G, 401F, 431L, 538K, 584S, 650N, 954D, 963S, 1142K, 1292N, 1600G
EOG666T1W	domeless	JAK/STAT pathway	1435	0.00390	0.02112	12Q, 21K, 43V, 45R, 227L, 252Y, 298K, 324N, 494S, 496V, 1183I, 1309V, 1337G
EOG68W9HQ	Clect–8	C–type lectin	220	0.00006	0.00098	**177E**, 180S, 181S, **183P**, **185S**, 188Q, **189Q**, 204T, 207E, 213Y, **216K**, **220N**
EOG6J3TZ2	cardinal	Peroxidase	1186	0.00011	0.00171	69V, 330K, 416S, 477S, 672G, 799K, 850R, 861N, 920N, 1072E, 1161S, 1186S
EOG6X0K8Q	myd88	Toll pathway	209	0.00334	0.01961	**1P**, 32E, 36K, 39T, 85N, **86E**, 87M, 142K, 146N, 175Q, **200S**
EOG6QRFJX	draper	Nimrod	600	0.00733	0.03042	**2Q**, **3E**, 133L, 217E, 227S, 296I, 325W, 330T, 336E, 346W
EOG64J10C	ird5	IMD pathway	343	0.00840	0.03383	1G, 41E, 46S, 142Q, 146G, 153N, 182S, 214Y, 285Y
EOG6FBG9K	npc2a	MD–2–related lipid recognition	138	0.00327	0.01961	3I, 6I, **10C**, 14P, 25T, 82Q, 85D, **113S**, 127D
EOG659ZWS	relish	Relish	609	0.00086	0.00793	**23S**, 167C, 178N, 181K, 342A, 452S, 461S, 552H
EOG62547K	armitage	Small RNA regulatory pathway members	996	0.00119	0.00984	5F, 143E, 415Q, 451S, 493I, 512I, 985G
EOG6R7SS0	CLIP–D3	CLIP serine protease	298	0.00068	0.00688	7S, 14Q, 19K, **20E**, **92E**, 120H
EOG6X3FGJ	SCR–B6	Scavenger receptor	438	0.00506	0.02378	106A, 195S, 196Y, 264I, 298K, 354V
EOG6TTF0B	spatzle–6	Spaetzle	385	0.00421	0.02197	21V, 152S, **153D**, **154S**, 157L, 381S
EOG66DJHQ	aubergine	Small RNA regulatory pathway members	644	0.00012	0.00171	25E, 325R, 353P, 627D, **633M**, 634Y
EOG6C2FSC	atg1	Autophagy	471	0.00165	0.01290	65R, 81H, 217C, 283M, 285A
EOG6RBP1C	persephone	CLIP serine protease	288	0.00255	0.01826	79I, 167A, 169N, 192V, 195P
EOG6DFN3J	PPO–6	Prophenol oxidase	672	0.00547	0.02410	**173E**, 226N, 375I, 421Q, **568G**
EOG6X0K76	tep3	Thioester–containing protein	301	0.00060	0.00653	**1P**, 9F, 10T, 13S, 127E
EOG680GDM	eiger	c–Jun N–terminal kinases	334	0.01119	0.04283	101L, 130K, 140N, 157S
EOG6N8PNJ	wengen	c–Jun N–terminal kinases	211	0.00020	0.00229	146S, **198E**, **206N**, 208I
EOG6866VT	tube	Toll pathway	88	0.01124	0.04283	25Q, 79Y, **82V**, 88F
EOG61VHHT	Clect–SE1	C–type lectin	852	0.00528	0.02402	30S, 35C, 86Y
EOG679CP4	POSH	IMD pathway	852	0.00289	0.01850	310Q, **311N**, 574Y
EOG62Z354	argonaute–3	Small RNA regulatory pathway members	743	0.00371	0.02094	81E, 130Q, 395G
EOG6WH726	atg6	Autophagy	430	0.00259	0.01826	**11A**, 26Y
EOG6NCJV4	snake	CLIP serine protease	399	0.00496	0.02378	66V, 78I
EOG6TX97R	CLIP–D1–like protein	CLIP serine protease	312	0.00278	0.01850	23C, 59Q
EOG6RV16R–2	BGRP–2	GNBP	234	0.00472	0.02377	123R, 224K
EOG64QRG8	tak1	c–Jun N–terminal kinases	150	0.00704	0.03006	43L, 44D
EOG66DJHX–1	dscam–like protein	Immunoglobulin	1495	0.00000	0.00000	None
EOG66DJHX–2	dscam	Immunoglobulin	489	0.00000	0.00000	None

[a] Group identifiers are from OrthoDB 6 (http://cegg.unige.ch/orthodb6).
[b] Unless otherwise specified, gene names are taken from the *A. mellifera* or *D. melanogaster* orthologs.
[c] Total number of codons in the alignment after trimming with Gblocks.
[d] From LRT comparing Branch–site model A to a constrained version with $\omega_2 = 1$.
[e] Multiple test correction by the method of Benjamini and Hochberg to control the false discovery rate.
[f] Sites are classified as under positive selection if the Bayesian posterior probability > 0.75 (> 0.95 in bold). Reference sequence taken from *A. mellifera*
