# Supplementary material for: A depauperate immune repertoire precedes evolution of sociality in bees
Source: Genome Biol. 2015 Apr 24;16(1):83. doi: 10.1186/s13059-015-0628-y (PMC4408586; doi:10.1186/s13059-015-0628-y)
Supplement: Additional file 8: — Genes that tested significant for evolving under differenct selective pressures (FDR < 0.05) between Bombus and Apis according to Clade model D (4 taxa tree). [file 13059_2015_628_MOESM8_ESM.pdf]

Genes that tested significant for evolving under different selective pressures (FDR < 0.05) between *Bombus* and *Apis*, according to Clade model D (4 taxa tree)

OrthoDB Group[a]	Gene[b]	Classification	Sites[c]	*p*–value[d]	*q*–value[e]	Proportion[f]
EOG6KKWJ6	fmr1	Small RNA regulatory pathway members	377	0.02746	0.04681	1.00000
EOG634TN0	drosha	Small RNA regulatory pathway members	1304	0.00911	0.01898	0.92316
EOG6Q2BWQ	IAP–2	IAP repeat	515	0.00082	0.00257	0.89698
EOG6W9GKN	atg7	Autophagy	668	0.02481	0.04278	0.81241
EOG680GC4	CLIP–D9	CLIP serine protease	503	0.02967	0.04838	0.78231
EOG6CNP6K–1	SCR–B3	Scavenger receptor	569	0.02200	0.03838	0.75704
EOG6J3TZ2	cardinal	Peroxidase	1203	0.00000	0.00000	0.61809
EOG6VX0M3	ark	Caspase	1263	0.00001	0.00005	0.55688
EOG69CNQ4–1	IAP–1A	IAP repeat	401	0.00000	0.00002	0.50305
EOG6XKSQD	peroxiredoxin–1	Peroxidase	208	0.00028	0.00115	0.49032
EOG6FBG95	Clect–10	C–type lectin	159	0.00214	0.00554	0.41115
EOG62Z354	argonaute–3	Small RNA regulatory pathway members	814	0.00000	0.00000	0.40856
EOG6RBP1B	cactus	Toll pathway	357	0.01524	0.02874	0.40496
EOG66DJHX–1	dscam–like protein	Immunoglobulin	1847	0.00013	0.00063	0.38471
EOG6BG79T	spindle–E	Small RNA regulatory pathway members	1273	0.00000	0.00000	0.35075
EOG6RBP1C	persephone	CLIP serine protease	293	0.00004	0.00021	0.34524
EOG6CNP6K–2	SCR–B9	Scavenger receptor	387	0.00034	0.00139	0.29157
EOG6QRFKP	CLIP–C1B	CLIP serine protease	350	0.00381	0.00908	0.26999
EOG6XWDCW	rm62–C	Small RNA regulatory pathway members	526	0.00000	0.00000	0.25556
EOG6VDNFR	dorsal	Relish	476	0.00000	0.00000	0.25203
EOG6RJDHD	r2d2	Small RNA regulatory pathway members	325	0.00360	0.00870	0.24607
EOG66DJHQ	aubergine	Small RNA regulatory pathway members	787	0.00000	0.00000	0.24311
EOG6XWDDG–2	serpin–9	Serine protease inhibitor	365	0.00000	0.00002	0.24101
EOG6TDZ3C	defensin	AMP	93	0.02861	0.04775	0.23445
EOG6ZPC96	nimrod–C2	Nimrod	1802	0.00000	0.00000	0.22238
EOG6QNKCB	spatzle–1B	Spaetzle	169	0.00002	0.00011	0.21683
EOG6D2556	atg4A	Autophagy	482	0.00000	0.00004	0.21463
EOG6X3FG7	tudor–SN	Small RNA regulatory pathway members	872	0.00008	0.00042	0.16680
EOG6VX0M1–2	DBLOX–like protein	Peroxidase	699	0.00001	0.00009	0.16364
EOG666T1W	domeless	JAK/STAT pathway	1490	0.00010	0.00048	0.16255
EOG6X3FGJ	SCR–B6	Scavenger receptor	437	0.00057	0.00197	0.15961
EOG6RV16R–2	BGRP–2	GNBP	322	0.01280	0.02526	0.15850
EOG61ZCRN	IAP–6	IAP repeat	3255	0.00000	0.00000	0.15842
EOG6FJ6RD	CLIP–B13	CLIP serine protease	355	0.00082	0.00257	0.13351
EOG6FN2ZK	hopscotch	JAK/STAT pathway	1088	0.00002	0.00009	0.13244
EOG6XWDDG–3	serpin–10B	Serine protease inhibitor	307	0.00047	0.00172	0.12278
EOG62547K	armitage	Small RNA regulatory pathway members	1054	0.02865	0.04775	0.11909
EOG634TMX	dicer–2	Small RNA regulatory pathway members	1334	0.00004	0.00023	0.11437
EOG6W9GK1–1	rm62–J	Small RNA regulatory pathway members	708	0.00038	0.00149	0.11130
EOG64F4RN	traf6	Toll pathway	409	0.02043	0.03649	0.10458
EOG6ZCRKS	loquacious	Small RNA regulatory pathway members	332	0.00001	0.00008	0.09908
EOG659ZWS	relish	Relish	613	0.00066	0.00226	0.09857
EOG693216–1	peroxiredoxin–4	Peroxidase	220	0.00043	0.00160	0.09726
EOG6866VT	tube	Toll pathway	298	0.00110	0.00309	0.09416
EOG6TX97R	CLIP–D1–like protein	CLIP serine protease	320	0.00050	0.00179	0.08981
EOG60GB73–1	PHGPx2B	Peroxidase	198	0.00204	0.00536	0.08765
EOG6T1G2P	pelle	Toll pathway	414	0.00000	0.00002	0.08323
EOG6BG7B9	snmp1	Scavenger receptor	484	0.00111	0.00309	0.08052
EOG68SF83	tep23	Thioester–containing protein	1694	0.00000	0.00000	0.07874
EOG62BVQX	caspase–L1	Caspase	396	0.00448	0.01003	0.07823
EOG6612K2	rm62–A	Small RNA regulatory pathway members	725	0.01434	0.02793	0.07771
EOG6ZCRN2	IAP–5	IAP repeat	145	0.02947	0.04838	0.07768
EOG6QRFJX	draper	Nimrod	945	0.00107	0.00309	0.07296
EOG6MGQPH	atg16L	Autophagy	160	0.00000	0.00000	0.05986
EOG6KWH93	vig	Small RNA regulatory pathway members	411	0.00641	0.01373	0.05947
EOG66DJHX–2	dscam	Immunoglobulin	777	0.00000	0.00000	0.05396
EOG6D254M	SCR–AC1	Scavenger receptor	2816	0.00000	0.00000	0.05324
EOG61NS2P	CLIP–A5	CLIP serine protease	308	0.00007	0.00037	0.05321
EOG6WDBSW	serpin–28D	Serine protease inhibitor	601	0.02155	0.03802	0.04934
EOG66Q57J	LOC100642902 (B. terr)	Serine protease inhibitor	1327	0.00014	0.00063	0.04831
EOG6PRR5Q	CLIP–A30	CLIP serine protease	875	0.00000	0.00003	0.04632
EOG6XWDDG–1	serpin–10A	Serine protease inhibitor	385	0.00419	0.00982	0.04073
EOG6VX0M1–1	DBLOX	Peroxidase	944	0.00630	0.01369	0.04017
EOG6J6Q5V	peroxidasin	Peroxidase	1100	0.00284	0.00704	0.03994
EOG6RJDH9	scarface	CLIP serine protease	622	0.00019	0.00083	0.03490
EOG6X0K8Q	myd88	Toll pathway	275	0.00000	0.00000	0.03275
EOG6ZPC9T	rm62–F	Small RNA regulatory pathway members	546	0.01087	0.02202	0.03193
EOG6VX0M4	rm62–I	Small RNA regulatory pathway members	713	0.00432	0.00982	0.03051
EOG6JSXPT	abaecin	AMP	46	0.00591	0.01303	0.02894
EOG6KKWHX	argonaute–2	Small RNA regulatory pathway members	896	0.00000	0.00000	0.02857
EOG6TTDZQ	dicer–1	Small RNA regulatory pathway members	1908	0.00120	0.00326	0.01967
EOG608KPT	LOC100642932 (B. terr)	Scavenger receptor	1893	0.00103	0.00304	0.01939
EOG61VHHT	Clect–SE1	C–type lectin	1095	0.00041	0.00158	0.01936
EOG69CNQ4–2	IAP–1B	IAP repeat	346	0.00709	0.01498	0.01823
EOG634TN8	croquemort	Scavenger receptor	487	0.00001	0.00008	0.01679
EOG6C2FSC	atg1	Autophagy	476	0.00092	0.00277	0.01352
EOG6W9GK1–3	rm62–B1	Small RNA regulatory pathway members	431	0.00080	0.00257	0.01244
EOG6X0K76	tep3	Thioester–containing protein	1379	0.00927	0.01904	0.01054
EOG6HDR8C	TLR–1	Toll receptor	496	0.00428	0.00982	0.01047
EOG64J10C	ird5	IMD pathway	560	0.00148	0.00395	0.00921
EOG6RV16R–1	BGRP–1	GNBP	457	0.00016	0.00072	0.00850
EOG6KD521	chorion peroxidase	Peroxidase	482	0.00086	0.00264	0.00770
EOG6W3R35	belle	Small RNA regulatory pathway members	683	0.01168	0.02335	0.00701
EOG6HHMH6	serpin–23	Scavenger receptor	2066	0.00008	0.00041	0.00664
EOG6JQ2CF	LOC100642575 (B. terr)	Scavenger receptor	924	0.01621	0.02966	0.00648
EOG6931ZS–1	TLR–7	Toll receptor	1344	0.00255	0.00647	0.00507
EOG6DFN3J	PPO–6	Prophenol oxidase	673	0.01621	0.02966	0.00495
EOG634TNR	pellino	Toll pathway	431	0.00078	0.00257	0.00483
EOG679CP4	POSH	IMD pathway	866	0.01504	0.02874	0.00461
EOG6N8PNJ	wengen	c–Jun N–terminal kinases	293	0.01533	0.02874	0.00449
EOG641NSQ	stat	JAK/STAT pathway	774	0.00286	0.00704	0.00283
EOG6N5TBN	galectin–3	Galectin	1290	0.01847	0.03338	0.00158

[a] Group identifiers are from OrthoDB 6 (http://cegg.unige.ch/orthodb6).
[b] Unless otherwise specified, gene names are taken from the *A. mellifera* or *D. melanogaster* orthologs.
[c] Total number of codons in the alignment after trimming with Gblocks.
[d] From LRT comparing Clade model D to M3 with 3 rate categories.
[e] Multiple test correction by the method of Benjamini and Hochberg to control the false discovery rate.
[f] Maximum likelihood estimate for the proportion of sites evolving differently between clades.
