# Supplementary material for: A depauperate immune repertoire precedes evolution of sociality in bees
Source: Genome Biol. 2015 Apr 24;16(1):83. doi: 10.1186/s13059-015-0628-y (PMC4408586; doi:10.1186/s13059-015-0628-y)
Supplement: Additional file 9: — Genes that tested significant for evolving under different selective pressures (FDR < 0.05) between the social and solitary clades according to Clade model D (5 taxa tree). [file 13059_2015_628_MOESM9_ESM.pdf]

Genes that tested significant for evolving under different selective pressures (FDR < 0.05) between the social and solitary clades, according to Clade model D (5 taxa tree)

OrthoDB Group[a]	Gene[b]	Classification	Sites[c]	*p*–value[d]	*q*–value[e]	Proportion[f]
EOG60001Z	atg8	Autophagy	117	0.01626	0.02667	1.00000
EOG679CPZ	spatzle–5	Spaetzle	222	0.00259	0.00609	0.75101
EOG6PC86T	tor	Autophagy	2317	0.03019	0.04627	0.65667
EOG6HDR8C	TLR–1	Toll receptor	376	0.00159	0.00416	0.52761
EOG6KD521	chorion peroxidase	Peroxidase	352	0.00000	0.00000	0.47206
EOG60GB73–1	PHGPx2B	Peroxidase	196	0.00001	0.00004	0.45950
EOG6RJDHD	r2d2	Small RNA regulatory pathway members	325	0.00026	0.00098	0.38934
EOG66DJHX–1	dscam–like protein	Immunoglobulin	1495	0.00912	0.01613	0.37982
EOG66WWRH–2	Clect–GA2	C–type lectin	169	0.00000	0.00004	0.37893
EOG6VX0NG–2	PGRP–S2	PRGP	111	0.01433	0.02424	0.37639
EOG6RBP1B	cactus	Toll pathway	356	0.00000	0.00001	0.37320
EOG66WWRH–1	Clect–GA1	C–type lectin	211	0.00038	0.00128	0.35494
EOG66DJHQ	aubergine	Small RNA regulatory pathway members	644	0.00000	0.00000	0.35278
EOG6VX0NG–1	PGRP–LF	PRGP	187	0.00019	0.00074	0.32989
EOG6X3FGJ	SCR–B6	Scavenger receptor	438	0.00013	0.00054	0.31159
EOG634TN8	croquemort	Scavenger receptor	477	0.00015	0.00061	0.29413
EOG6J3TZ2	cardinal	Peroxidase	1186	0.00000	0.00000	0.28417
EOG6TX97R	CLIP–D1–like protein	CLIP serine protease	312	0.00038	0.00128	0.27317
EOG6QRFKP	CLIP–C1B	CLIP serine protease	330	0.00001	0.00004	0.25991
EOG6W9GK1–1	rm62–J	Small RNA regulatory pathway members	678	0.01019	0.01753	0.25007
EOG6FJ6RD	CLIP–B13	CLIP serine protease	346	0.00719	0.01389	0.23045
EOG6FBG9K	npc2a	MD–2–related lipid recognition	138	0.00009	0.00041	0.22516
EOG6XWDDG–1	serpin–10A	Serine protease inhibitor	375	0.00000	0.00000	0.22037
EOG6W9GK1–3	rm62–B1	Small RNA regulatory pathway members	405	0.00915	0.01613	0.21828
EOG64B8H5	CLIP–A10	CLIP serine protease	792	0.01990	0.03188	0.20892
EOG6CNP6K–2	SCR–B9	Scavenger receptor	387	0.00001	0.00007	0.19529
EOG61C5BT	spatzle–3	Spaetzle	393	0.00268	0.00612	0.18193
EOG6WDBSW	serpin–28D	Serine protease inhibitor	423	0.00190	0.00479	0.17266
EOG6DV43B	immune deficiency	IMD pathway	249	0.00463	0.00960	0.16254
EOG64J10C	ird5	IMD pathway	343	0.00269	0.00612	0.15100
EOG6D254M	SCR–AC1	Scavenger receptor	1650	0.00000	0.00000	0.13877
EOG6DFN3J	PPO–6	Prophenol oxidase	672	0.00002	0.00009	0.13747
EOG6NCJV4	snake	CLIP serine protease	399	0.00392	0.00824	0.13438
EOG6BG79T	spindle–E	Small RNA regulatory pathway members	1268	0.00189	0.00479	0.13093
EOG6K6DKF	cactin	Toll pathway	559	0.00000	0.00000	0.13066
EOG6RBP1C	persephone	CLIP serine protease	288	0.00135	0.00374	0.11960
EOG66Q57J	LOC100642902 (B. terr)	Serine protease inhibitor	1189	0.00000	0.00000	0.11842
EOG69W0XF	ran	Small RNA regulatory pathway members	127	0.00299	0.00659	0.11155
EOG6J6Q5V	peroxidasin	Peroxidase	1091	0.00013	0.00054	0.10978
EOG6BG7B9	snmp1	Scavenger receptor	430	0.00005	0.00025	0.10927
EOG64QRG8	tak1	c–Jun N–terminal kinases	150	0.00863	0.01580	0.10700
EOG61ZCRN	IAP–6	IAP repeat	2053	0.00000	0.00001	0.10601
EOG68W9HQ	Clect–8	C–type lectin	220	0.00000	0.00000	0.10533
EOG634TPR	galectin–5	Galectin	416	0.01020	0.01753	0.10333
EOG6NVX17	argonaute–1	Small RNA regulatory pathway members	813	0.00000	0.00001	0.10250
EOG6X0K8Q	myd88	Toll pathway	209	0.00029	0.00108	0.09836
EOG66T1GW	caspar	IMD pathway	597	0.00101	0.00303	0.09006
EOG6CRJF5	masquerade	CLIP serine protease	146	0.00054	0.00166	0.08448
EOG634TMW	ranbp–21	Small RNA regulatory pathway members	1213	0.01444	0.02424	0.08406
EOG6ZPC9T	rm62–F	Small RNA regulatory pathway members	545	0.00152	0.00412	0.07861
EOG6MGQPH	atg16L	Autophagy	159	0.02075	0.03288	0.07850
EOG6VDNFR	dorsal	Relish	353	0.00249	0.00596	0.07499
EOG634TMX	dicer–2	Small RNA regulatory pathway members	1269	0.00000	0.00000	0.07494
EOG6Q2BWQ	IAP–2	IAP repeat	503	0.00000	0.00000	0.07304
EOG6KKWHX	argonaute–2	Small RNA regulatory pathway members	810	0.00857	0.01580	0.07167
EOG6866VT	tube	Toll pathway	88	0.00156	0.00416	0.06780
EOG6QRFJX	draper	Nimrod	600	0.00000	0.00001	0.06624
EOG659ZWS	relish	Relish	609	0.00001	0.00005	0.06560
EOG680GDM	eiger	c–Jun N–terminal kinases	334	0.00113	0.00318	0.06252
EOG6PRR5Q	CLIP–A30	CLIP serine protease	812	0.00211	0.00514	0.06208
EOG6XKSQD	peroxiredoxin–1	Peroxidase	208	0.01562	0.02591	0.06120
EOG680GC4	CLIP–D9	CLIP serine protease	405	0.00001	0.00007	0.05663
EOG6R7SS0	CLIP–D3	CLIP serine protease	298	0.00011	0.00051	0.05254
EOG6931ZS–3	TLR–6	Toll receptor	1217	0.00047	0.00146	0.05198
EOG6FN2ZK	hopscotch	JAK/STAT pathway	600	0.00000	0.00000	0.05174
EOG6HX3GG	stubble	CLIP serine protease	660	0.00626	0.01243	0.04943
EOG6612K2	rm62–A	Small RNA regulatory pathway members	726	0.02569	0.03981	0.04925
EOG6KWH93	vig	Small RNA regulatory pathway members	411	0.00111	0.00318	0.04554
EOG6VX0M1–1	DBLOX	Peroxidase	932	0.00707	0.01385	0.04195
EOG6RV16R–2	BGRP–2	GNBP	234	0.00534	0.01092	0.04005
EOG6N8PNJ	wengen	c–Jun N–terminal kinases	211	0.00006	0.00027	0.03808
EOG6X0K76	tep3	Thioester–containing protein	301	0.00002	0.00013	0.03382
EOG64B8HP	kayak	c–Jun N–terminal kinases	184	0.00359	0.00768	0.03281
EOG66DJHX–2	dscam	Immunoglobulin	489	0.00000	0.00000	0.03225
EOG62547K	armitage	Small RNA regulatory pathway members	996	0.00030	0.00108	0.03220
EOG6W9GKN	atg7	Autophagy	667	0.00200	0.00495	0.02937
EOG608KPT	LOC100642932 (B. terr)	Scavenger receptor	1646	0.00000	0.00000	0.02900
EOG6W6MC0	Clect–GA3	C–type lectin	230	0.00046	0.00146	0.02770
EOG6JQ2CF	LOC100642575 (B. terr)	Scavenger receptor	744	0.00045	0.00146	0.02633
EOG6TTF0B	spatzle–6	Spaetzle	385	0.00001	0.00005	0.02575
EOG6C2FSC	atg1	Autophagy	471	0.00299	0.00659	0.02303
EOG6TTDZQ	dicer–1	Small RNA regulatory pathway members	1846	0.00820	0.01562	0.02229
EOG6GXD37	pasha	Small RNA regulatory pathway members	369	0.00593	0.01195	0.01951
EOG6931ZS–2	TLR–10	Toll receptor	1204	0.00002	0.00013	0.01744
EOG6X95Z0	tepA	Thioester–containing protein	1559	0.00839	0.01577	0.01413
EOG6VX0M1–2	DBLOX–like protein	Peroxidase	502	0.02493	0.03905	0.01397
EOG666T1W	domeless	JAK/STAT pathway	1435	0.00036	0.00128	0.01358
EOG6WH726	atg6	Autophagy	430	0.00019	0.00074	0.00922
EOG61VHHT	Clect–SE1	C–type lectin	852	0.00311	0.00675	0.00744
EOG679CP4	POSH	IMD pathway	852	0.00104	0.00305	0.00667
EOG641NSQ	stat	JAK/STAT pathway	736	0.01989	0.03188	0.00644
EOG6931ZS–1	TLR–7	Toll receptor	1299	0.00909	0.01613	0.00572

[a] Group identifiers are from OrthoDB 6 (http://cegg.unige.ch/orthodb6).
[b] Unless otherwise specified, gene names are taken from the *A. mellifera* or *D. melanogaster* orthologs.
[c] Total number of codons in the alignment after trimming with Gblocks.
[d] From LRT comparing Clade model D to M3 with 3 rate categories.
[e] Multiple test correction by the method of Benjamini and Hochberg to control the false discovery rate.
[f] Maximum likelihood estimate for the proportion of sites evolving differently between clades.
