# Supplementary material for: A depauperate immune repertoire precedes evolution of sociality in bees
Source: Genome Biol. 2015 Apr 24;16(1):83. doi: 10.1186/s13059-015-0628-y (PMC4408586; doi:10.1186/s13059-015-0628-y)
Supplement: Additional file 10: — Summary of models on the 4 taxa tree. [file 13059_2015_628_MOESM10_ESM.pdf]

Summary of models on the 4 taxa tree.

OrthoDB Group[a]	Gene[b]	Classification	Sites[c]	Global ω[1]	M8 vs M7[2]	Branch–site[3]	Clade D[4]
EOG6JSXPT	abaecin	AMP	46	0.33584			X
EOG6TDZ3C	defensin	AMP	93	0.2081			X
EOG6C2FSC	atg1	Autophagy	476	0.13269			X
EOG644J2Q	atg10	Autophagy	162	0.11878			
EOG6MGQPH	atg16L	Autophagy	160	0.12256			X
EOG6DR7V8	atg18	Autophagy	338	0.01356			
EOG69KD5B	atg2	Autophagy	1983	0.07114			
EOG6D2556	atg4A	Autophagy	482	0.06251			X
EOG602V84	atg4B	Autophagy	371	0.05558			
EOG6P8D0V	atg5	Autophagy	265	0.02194			
EOG6WH726	atg6	Autophagy	430	0.01014			
EOG6W9GKN	atg7	Autophagy	668	0.08236			X
EOG60001Z	atg8	Autophagy	117	1e–04			
EOG6Q574K	atg9	Autophagy	836	0.07233			
EOG6NP5KW	buffy	Autophagy	270	0.06015			
EOG6PC86T	tor	Autophagy	2407	0.01111			
EOG6XPNXB	basket	c–Jun N–terminal kinases	388	0.02014			
EOG680GDM	eiger	c–Jun N–terminal kinases	374	0.12769			
EOG61893B	hemipterous	c–Jun N–terminal kinases	759	0.03338			
EOG6K3JCB	jra	c–Jun N–terminal kinases	196	0.27614			
EOG64B8HP	kayak	c–Jun N–terminal kinases	185	0.019			
EOG64QRG8	tak1	c–Jun N–terminal kinases	407	0.11524			
EOG6N8PNJ	wengen	c–Jun N–terminal kinases	293	0.18255			X
EOG6FBG95	Clect–10	C–type lectin	159	0.14616			X
EOG68W9HQ	Clect–8	C–type lectin	412	0.07825			
EOG66WWRH–1	Clect–GA1	C–type lectin	217	0.02995			
EOG66WWRH–2	Clect–GA2	C–type lectin	216	0.04598			
EOG6W6MC0	Clect–GA3	C–type lectin	230	0.02812			
EOG61VHHT	Clect–SE1	C–type lectin	1095	0.03638			X
EOG6612JX	Clect–SE2	C–type lectin	866	0.06072			
EOG6VX0M3	ark	Caspase	1263	0.1294	X		X
EOG62BVQX	caspase–L1	Caspase	396	0.16633			X
EOG6F1VKC	ice	Caspase	297	0.12397			
EOG6Z8WBN	catalase	Catalase	221	0.10103			
EOG64B8H5	CLIP–A10	CLIP serine protease	816	0.12855	X		
EOG6PRR5Q	CLIP–A30	CLIP serine protease	875	0.1305			X
EOG61NS2P	CLIP–A5	CLIP serine protease	308	0.2122			X
EOG6FJ6RD	CLIP–B13	CLIP serine protease	355	0.26853			X
EOG6QRFKP	CLIP–C1B	CLIP serine protease	350	0.3294			X
EOG6TX97G	CLIP–D1	CLIP serine protease	241	0.22975			
EOG6TX97R	CLIP–D1–like protein	CLIP serine protease	320	0.36655			X
EOG6GHX4B	CLIP–D10	CLIP serine protease	308	0.00845			
EOG6R7SS0	CLIP–D3	CLIP serine protease	569	0.09458			
EOG680GC4	CLIP–D9	CLIP serine protease	503	0.08069			X
EOG6CRJF5	masquerade	CLIP serine protease	603	0.09003			
EOG6RBP1C	persephone	CLIP serine protease	293	0.27289			X
EOG6RJDH9	scarface	CLIP serine protease	622	0.30015			X
EOG6NCJV4	snake	CLIP serine protease	404	0.26193			
EOG6HX3GG	stubble	CLIP serine protease	695	0.06682			
EOG6N5TBN	galectin–3	Galectin	1290	0.0529			X
EOG634TPR	galectin–5	Galectin	423	0.10772			
EOG6RV16R–1	BGRP–1	GNBP	457	0.2749			X
EOG6RV16R–2	BGRP–2	GNBP	322	0.22745			X
EOG69CNQ4–1	IAP–1A	IAP repeat	401	0.14696			X
EOG69CNQ4–2	IAP–1B	IAP repeat	346	0.24138			X
EOG6Q2BWQ	IAP–2	IAP repeat	515	0.14886			X
EOG6ZCRN2	IAP–5	IAP repeat	145	0.09251			X
EOG61ZCRN	IAP–6	IAP repeat	3255	0.04459			X
EOG66T1GW	caspar	IMD pathway	614	0.03108			
EOG6ZW3V0	fadd	IMD pathway	144	0.22926			
EOG6DV43B	immune deficiency	IMD pathway	256	0.20362			
EOG64J10C	ird5	IMD pathway	560	0.19261			X
EOG679CP4	POSH	IMD pathway	866	0.05316			X
EOG66DJHX–2	dscam	Immunoglobulin	777	0.05496	X		X
EOG66DJHX–1	dscam–like protein	Immunoglobulin	1847	0.02561		X	X
EOG666T1W	domeless	JAK/STAT pathway	1490	0.10803			X
EOG6FN2ZK	hopscotch	JAK/STAT pathway	1088	0.10446			X
EOG641NSQ	stat	JAK/STAT pathway	774	0.01621			X
EOG6FBG9K	npc2a	MD–2–related lipid recognition	149	0.17968			
EOG6NVX30	npc2b	MD–2–related lipid recognition	127	0.00435			
EOG6QRFJX	draper	Nimrod	945	0.16982			X
EOG6ZPC96	nimrod–C2	Nimrod	1802	0.12496		X	X
EOG6J3TZ2	cardinal	Peroxidase	1203	0.08436	X		X
EOG6KD521	chorion peroxidase	Peroxidase	482	0.0574			X
EOG6VX0M1–1	DBLOX	Peroxidase	944	0.01738			X
EOG6VX0M1–2	DBLOX–like protein	Peroxidase	699	0.04801			X
EOG6J9KDP	Duox	Peroxidase	1458	0.18854			
EOG6J6Q5V	peroxidasin	Peroxidase	1100	0.07237			X
EOG6XKSQD	peroxiredoxin–1	Peroxidase	208	0.05024			X
EOG693216–1	peroxiredoxin–4	Peroxidase	220	0.12329			X
EOG693216–2	peroxiredoxin–5	Peroxidase	220	0.05527			
EOG60GB73–2	PHGPx2A	Peroxidase	166	0.09657			
EOG60GB73–1	PHGPx2B	Peroxidase	198	0.2249			X
EOG6VX0NG–1	PGRP–LF	PRGP	190	0.06285			
EOG6VX0NG–3	PGRP–S1	PRGP	103	0.11065			
EOG6VX0NG–2	PGRP–S2	PRGP	117	0.16311			
EOG6DFN3J	PPO–6	Prophenol oxidase	673	0.19318			X
EOG6VDNFR	dorsal	Relish	476	0.05633		X	X
EOG659ZWS	relish	Relish	613	0.109			X
EOG634TN8	croquemort	Scavenger receptor	487	0.17554			X
EOG6JQ2CF	LOC100642575 (B. terr)	Scavenger receptor	924	0.07198	X		X
EOG608KPT	LOC100642932 (B. terr)	Scavenger receptor	1893	0.11229			X
EOG6D254M	SCR–AC1	Scavenger receptor	2816	0.08879			X
EOG6CNP6K–1	SCR–B3	Scavenger receptor	569	0.16667			X
EOG6W0VV6	SCR–B5	Scavenger receptor	494	0.21578			
EOG6X3FGJ	SCR–B6	Scavenger receptor	437	0.07868			X
EOG6CNP6K–2	SCR–B9	Scavenger receptor	387	0.11332			X
EOG6HHMH6	serpin–23	Scavenger receptor	2066	0.09921	X	X	X
EOG6BG7B9	snmp1	Scavenger receptor	484	0.1691			X
EOG66Q57J	LOC100642902 (B. terr)	Serine protease inhibitor	1327	0.29567		X	X
EOG6XWDDG–1	serpin–10A	Serine protease inhibitor	385	0.21958		X	X
EOG6XWDDG–3	serpin–10B	Serine protease inhibitor	307	0.17517			X
EOG6M37R0	serpin–27A	Serine protease inhibitor	406	0.03255			
EOG6WDBSW	serpin–28D	Serine protease inhibitor	601	0.2977			X
EOG6XWDDG–2	serpin–9	Serine protease inhibitor	365	0.25778			X
EOG6NVX17	argonaute–1	Small RNA regulatory pathway members	886	0.02329			
EOG6KKWHX	argonaute–2	Small RNA regulatory pathway members	896	0.12628	X	X	X
EOG62Z354	argonaute–3	Small RNA regulatory pathway members	814	0.21295			X
EOG62547K	armitage	Small RNA regulatory pathway members	1054	0.14429			X
EOG66DJHQ	aubergine	Small RNA regulatory pathway members	787	0.03453	X		X
EOG6W3R35	belle	Small RNA regulatory pathway members	683	0.05737		X	X
EOG6TTDZQ	dicer–1	Small RNA regulatory pathway members	1908	0.01077			X
EOG634TMX	dicer–2	Small RNA regulatory pathway members	1334	0.16362			X
EOG634TN0	drosha	Small RNA regulatory pathway members	1304	0.02405			X
EOG6KKWJ6	fmr1	Small RNA regulatory pathway members	377	0.02037			X
EOG6ZCRKS	loquacious	Small RNA regulatory pathway members	332	0.03403			X
EOG6GXD37	pasha	Small RNA regulatory pathway members	638	0.02071			
EOG6RJDHD	r2d2	Small RNA regulatory pathway members	325	0.05218			X
EOG69W0XF	ran	Small RNA regulatory pathway members	128	0.02393			
EOG634TMW	ranbp–21	Small RNA regulatory pathway members	1217	0.11061			
EOG6612K2	rm62–A	Small RNA regulatory pathway members	725	0.14078			X
EOG6W9GK1–3	rm62–B1	Small RNA regulatory pathway members	431	0.03383	X		X
EOG6XWDCW	rm62–C	Small RNA regulatory pathway members	526	0.02738			X
EOG6ZPC9T	rm62–F	Small RNA regulatory pathway members	546	0.06871			X
EOG6W9GK1–2	rm62–H	Small RNA regulatory pathway members	549	0.05434			
EOG6VX0M4	rm62–I	Small RNA regulatory pathway members	713	0.22813			X
EOG6W9GK1–1	rm62–J	Small RNA regulatory pathway members	708	0.00691			X
EOG6W9GK1–4	rm62–J1	Small RNA regulatory pathway members	402	0.01879			
EOG6BG79T	spindle–E	Small RNA regulatory pathway members	1273	0.25716		X	X
EOG6X3FG7	tudor–SN	Small RNA regulatory pathway members	872	0.03453			X
EOG6KWH93	vig	Small RNA regulatory pathway members	411	0.22524			X
EOG6QNKCB	spatzle–1B	Spaetzle	169	0.05277		X	X
EOG61C5BT	spatzle–3	Spaetzle	401	0.17389			
EOG679CPZ	spatzle–5	Spaetzle	277	0.183			
EOG6TTF0B	spatzle–6	Spaetzle	395	0.18222			
EOG68SF83	tep23	Thioester–containing protein	1694	0.09047		X	X
EOG6X0K76	tep3	Thioester–containing protein	1379	0.10134			X
EOG6X95Z0	tepA	Thioester–containing protein	1585	0.15891			
EOG6K6DKF	cactin	Toll pathway	569	0.11892			
EOG6RBP1B	cactus	Toll pathway	357	0.22611			X
EOG6X0K8Q	myd88	Toll pathway	275	0.21105			X
EOG6T1G2P	pelle	Toll pathway	414	0.04759			X
EOG634TNR	pellino	Toll pathway	431	0.02026	X		X
EOG64F4RN	traf6	Toll pathway	409	0.31941			X
EOG6866VT	tube	Toll pathway	298	0.01657		X	X
EOG6HDR8C	TLR–1	Toll receptor	496	0.03138			X
EOG6931ZS–2	TLR–10	Toll receptor	1280	0.04607			
EOG6931ZS–3	TLR–6	Toll receptor	1217	0.03131			
EOG6931ZS–1	TLR–7	Toll receptor	1344	0.08263			X
EOG6931ZS–4	TLR–8	Toll receptor	1184	0.02863			

[a] Group identifiers are from OrthoDB 6 (http://cegg.unige.ch/orthodb6).
[b] Unless otherwise specified, gene names are taken from the *A. mellifera* or *D. melanogaster* orthologs.
[c] Total number of codons in the alignment after trimming with Gblocks.

[1] Across the whole phylogeny using M0 model.
[2] Positive selection across the whole phylogeny.
[3] Positive selection on the branch between *Bombus* and *Apis*.
[4] Different selective pressures between *Bombus* and *Apis*.

Using FDR < 0.05 on all tests.
