# Supplementary material for: A depauperate immune repertoire precedes evolution of sociality in bees
Source: Genome Biol. 2015 Apr 24;16(1):83. doi: 10.1186/s13059-015-0628-y (PMC4408586; doi:10.1186/s13059-015-0628-y)
Supplement: Additional file 11: — Summary of models on the 5 taxa tree. [file 13059_2015_628_MOESM11_ESM.pdf]

Summary of models on the 5 taxa tree.

OrthoDB Group[a]	Gene[b]	Classification	Sites[c]	Global ω[1]	M8 vs M7[2]	Branch–site[3]	Branch–site[4]	Branch–site[5]	Branch–site[6]	Clade D[7]
EOG6JSXPT	abaecin	AMP	N/A	N/A						
EOG6TDZ3C	defensin	AMP	92	0.17525						
EOG6C2FSC	atg1	Autophagy	471	0.12007					X	X
EOG644J2Q	atg10	Autophagy	N/A	N/A						
EOG6MGQPH	atg16L	Autophagy	159	0.09132						X
EOG6DR7V8	atg18	Autophagy	262	0.03276						
EOG69KD5B	atg2	Autophagy	1775	0.07692						
EOG6D2556	atg4A	Autophagy	431	0.04811						
EOG602V84	atg4B	Autophagy	337	0.05112						
EOG6P8D0V	atg5	Autophagy	261	0.0265						
EOG6WH726	atg6	Autophagy	430	0.01267					X	X
EOG6W9GKN	atg7	Autophagy	667	0.08185						X
EOG60001Z	atg8	Autophagy	117	0.02379						X
EOG6Q574K	atg9	Autophagy	809	0.07441						
EOG6NP5KW	buffy	Autophagy	253	0.04091						
EOG6PC86T	tor	Autophagy	2317	0.00972						X
EOG6XPNXB	basket	c–Jun N–terminal kinases	388	0.00469						
EOG680GDM	eiger	c–Jun N–terminal kinases	334	0.19204					X	X
EOG61893B	hemipterous	c–Jun N–terminal kinases	761	0.03714						
EOG6K3JCB	jra	c–Jun N–terminal kinases	196	0.01599						
EOG64B8HP	kayak	c–Jun N–terminal kinases	184	0.02866						X
EOG64QRG8	tak1	c–Jun N–terminal kinases	150	0.2218					X	X
EOG6N8PNJ	wengen	c–Jun N–terminal kinases	211	0.18343					X	X
EOG6FBG95	Clect–10	C–type lectin	155	0.14098						
EOG68W9HQ	Clect–8	C–type lectin	220	0.07941					X	X
EOG66WWRH–1	Clect–GA1	C–type lectin	211	0.02727						X
EOG66WWRH–2	Clect–GA2	C–type lectin	169	0.04701						X
EOG6W6MC0	Clect–GA3	C–type lectin	230	0.03912						X
EOG61VHHT	Clect–SE1	C–type lectin	852	0.03676					X	X
EOG6612JX	Clect–SE2	C–type lectin	788	0.05268						
EOG6VX0M3	ark	Caspase	1128	0.11986	X		X	X		
EOG62BVQX	caspase–L1	Caspase	344	0.15962						
EOG6F1VKC	ice	Caspase	238	0.09912						
EOG6Z8WBN	catalase	Catalase	181	0.1076			X			
EOG64B8H5	CLIP–A10	CLIP serine protease	792	0.1226	X					X
EOG6PRR5Q	CLIP–A30	CLIP serine protease	812	0.10612						X
EOG61NS2P	CLIP–A5	CLIP serine protease	N/A	N/A						
EOG6FJ6RD	CLIP–B13	CLIP serine protease	346	0.22864						X
EOG6QRFKP	CLIP–C1B	CLIP serine protease	330	0.24449	X					X
EOG6TX97G	CLIP–D1	CLIP serine protease	201	0.17348						
EOG6TX97R	CLIP–D1–like protein	CLIP serine protease	312	0.30094					X	X
EOG6GHX4B	CLIP–D10	CLIP serine protease	294	0.01051						
EOG6R7SS0	CLIP–D3	CLIP serine protease	298	0.08745					X	X
EOG680GC4	CLIP–D9	CLIP serine protease	405	0.10056						X
EOG6CRJF5	masquerade	CLIP serine protease	146	0.01809						X
EOG6RBP1C	persephone	CLIP serine protease	288	0.24712					X	X
EOG6RJDH9	scarface	CLIP serine protease	378	0.25097						
EOG6NCJV4	snake	CLIP serine protease	399	0.23339					X	X
EOG6HX3GG	stubble	CLIP serine protease	660	0.06449						X
EOG6N5TBN	galectin–3	Galectin	1280	0.04932						
EOG634TPR	galectin–5	Galectin	416	0.12109						X
EOG6RV16R–1	BGRP–1	GNBP	459	0.24922			X	X		
EOG6RV16R–2	BGRP–2	GNBP	234	0.18544					X	X
EOG69CNQ4–1	IAP–1A	IAP repeat	N/A	N/A						
EOG69CNQ4–2	IAP–1B	IAP repeat	N/A	N/A						
EOG6Q2BWQ	IAP–2	IAP repeat	503	0.1256					X	X
EOG6ZCRN2	IAP–5	IAP repeat	139	0.09978						
EOG61ZCRN	IAP–6	IAP repeat	2053	0.05545						X
EOG66T1GW	caspar	IMD pathway	597	0.04391						X
EOG6ZW3V0	fadd	IMD pathway	134	0.20183						
EOG6DV43B	immune deficiency	IMD pathway	249	0.13835				X		X
EOG64J10C	ird5	IMD pathway	343	0.18295					X	X
EOG679CP4	POSH	IMD pathway	852	0.05525					X	X
EOG66DJHX–2	dscam	Immunoglobulin	489	0.05362	X				X	X
EOG66DJHX–1	dscam–like protein	Immunoglobulin	1495	0.02775					X	X
EOG666T1W	domeless	JAK/STAT pathway	1435	0.1038			X		X	X
EOG6FN2ZK	hopscotch	JAK/STAT pathway	600	0.08975					X	X
EOG641NSQ	stat	JAK/STAT pathway	736	0.01591						X
EOG6FBG9K	npc2a	MD–2–related lipid recognition	138	0.16468					X	X
EOG6NVX30	npc2b	MD–2–related lipid recognition	127	0.16092						
EOG6QRFJX	draper	Nimrod	600	0.0648					X	X
EOG6ZPC96	nimrod–C2	Nimrod	N/A	N/A						
EOG6J3TZ2	cardinal	Peroxidase	1186	0.15252					X	X
EOG6KD521	chorion peroxidase	Peroxidase	352	0.03149					X	X
EOG6VX0M1–1	DBLOX	Peroxidase	932	0.04732						X
EOG6VX0M1–2	DBLOX–like protein	Peroxidase	502	0.11747						X
EOG6J9KDP	Duox	Peroxidase	1431	0.0144						
EOG6J6Q5V	peroxidasin	Peroxidase	1091	0.0514						X
EOG6XKSQD	peroxiredoxin–1	Peroxidase	208	0.06932						X
EOG693216–1	peroxiredoxin–4	Peroxidase	220	0.06951						
EOG693216–2	peroxiredoxin–5	Peroxidase	220	0.05698						
EOG60GB73–2	PHGPx2A	Peroxidase	166	0.03937						
EOG60GB73–1	PHGPx2B	Peroxidase	196	0.08592						X
EOG6VX0NG–1	PGRP–LF	PRGP	187	0.09501						X
EOG6VX0NG–3	PGRP–S1	PRGP	76	0.13791						
EOG6VX0NG–2	PGRP–S2	PRGP	111	0.14224						X
EOG6DFN3J	PPO–6	Prophenol oxidase	672	0.07601					X	X
EOG6VDNFR	dorsal	Relish	353	0.1068		X	X			X
EOG659ZWS	relish	Relish	609	0.10713					X	X
EOG634TN8	croquemort	Scavenger receptor	477	0.14923						X
EOG6JQ2CF	LOC100642575 (B. terr)	Scavenger receptor	744	0.07663						X
EOG608KPT	LOC100642932 (B. terr)	Scavenger receptor	1646	0.13449					X	X
EOG6D254M	SCR–AC1	Scavenger receptor	1650	0.06841						X
EOG6CNP6K–1	SCR–B3	Scavenger receptor	500	0.07891						
EOG6W0VV6	SCR–B5	Scavenger receptor	476	0.16861						
EOG6X3FGJ	SCR–B6	Scavenger receptor	438	0.15942					X	X
EOG6CNP6K–2	SCR–B9	Scavenger receptor	387	0.06656						X
EOG6HHMH6	serpin–23	Scavenger receptor	650	0.19767						
EOG6BG7B9	snmp1	Scavenger receptor	430	0.10943			X			X
EOG66Q57J	LOC100642902 (B. terr)	Serine protease inhibitor	1189	0.24741		X	X	X	X	X
EOG6XWDDG–1	serpin–10A	Serine protease inhibitor	375	0.16206					X	X
EOG6XWDDG–3	serpin–10B	Serine protease inhibitor	307	0.2028						
EOG6M37R0	serpin–27A	Serine protease inhibitor	N/A	N/A						
EOG6WDBSW	serpin–28D	Serine protease inhibitor	423	0.19085						X
EOG6XWDDG–2	serpin–9	Serine protease inhibitor	337	0.11636						
EOG6NVX17	argonaute–1	Small RNA regulatory pathway members	813	0.01837						X
EOG6KKWHX	argonaute–2	Small RNA regulatory pathway members	810	0.22158	X	X	X			X
EOG62Z354	argonaute–3	Small RNA regulatory pathway members	743	0.11048					X	
EOG62547K	armitage	Small RNA regulatory pathway members	996	0.09973					X	X
EOG66DJHQ	aubergine	Small RNA regulatory pathway members	644	0.17942					X	X
EOG6W3R35	belle	Small RNA regulatory pathway members	647	0.04838						
EOG6TTDZQ	dicer–1	Small RNA regulatory pathway members	1846	0.05742						X
EOG634TMX	dicer–2	Small RNA regulatory pathway members	1269	0.13311					X	X
EOG634TN0	drosha	Small RNA regulatory pathway members	1290	0.03406				X		
EOG6KKWJ6	fmr1	Small RNA regulatory pathway members	343	0.02706						
EOG6ZCRKS	loquacious	Small RNA regulatory pathway members	332	0.07531						
EOG6GXD37	pasha	Small RNA regulatory pathway members	369	0.0386						X
EOG6RJDHD	r2d2	Small RNA regulatory pathway members	325	0.20087						X
EOG69W0XF	ran	Small RNA regulatory pathway members	127	0.00899						X
EOG634TMW	ranbp–21	Small RNA regulatory pathway members	1213	0.02043						X
EOG6612K2	rm62–A	Small RNA regulatory pathway members	726	0.01941						X
EOG6W9GK1–3	rm62–B1	Small RNA regulatory pathway members	405	0.02174						X
EOG6XWDCW	rm62–C	Small RNA regulatory pathway members	492	0.0699				X		
EOG6ZPC9T	rm62–F	Small RNA regulatory pathway members	545	0.09114			X			X
EOG6W9GK1–2	rm62–H	Small RNA regulatory pathway members	482	0.01533						
EOG6VX0M4	rm62–I	Small RNA regulatory pathway members	712	0.00638						
EOG6W9GK1–1	rm62–J	Small RNA regulatory pathway members	678	0.02782						X
EOG6W9GK1–4	rm62–J1	Small RNA regulatory pathway members	353	0.037						
EOG6BG79T	spindle–E	Small RNA regulatory pathway members	1268	0.11096						X
EOG6X3FG7	tudor–SN	Small RNA regulatory pathway members	772	0.02586						
EOG6KWH93	vig	Small RNA regulatory pathway members	411	0.05636						X
EOG6QNKCB	spatzle–1B	Spaetzle	N/A	N/A						
EOG61C5BT	spatzle–3	Spaetzle	393	0.06832						X
EOG679CPZ	spatzle–5	Spaetzle	222	0.12895						X
EOG6TTF0B	spatzle–6	Spaetzle	385	0.03401					X	X
EOG68SF83	tep23	Thioester–containing protein	N/A	N/A						
EOG6X0K76	tep3	Thioester–containing protein	301	0.0597					X	X
EOG6X95Z0	tepA	Thioester–containing protein	1559	0.04577						X
EOG6K6DKF	cactin	Toll pathway	559	0.03088						X
EOG6RBP1B	cactus	Toll pathway	356	0.17196						X
EOG6X0K8Q	myd88	Toll pathway	209	0.10847			X		X	X
EOG6T1G2P	pelle	Toll pathway	381	0.11016						
EOG634TNR	pellino	Toll pathway	428	0.01095						
EOG64F4RN	traf6	Toll pathway	363	0.10241						
EOG6866VT	tube	Toll pathway	88	0.11664					X	X
EOG6HDR8C	TLR–1	Toll receptor	376	0.25801						X
EOG6931ZS–2	TLR–10	Toll receptor	1204	0.03589						X
EOG6931ZS–3	TLR–6	Toll receptor	1217	0.03869						X
EOG6931ZS–1	TLR–7	Toll receptor	1299	0.02839			X			X
EOG6931ZS–4	TLR–8	Toll receptor	1177	0.01807						

[a] Group identifiers are from OrthoDB 6 (http://cegg.unige.ch/orthodb6).
[b] Unless otherwise specified, gene names are taken from the *A. mellifera* or *D. melanogaster* orthologs.
[c] Total number of codons in the alignment after trimming with Gblocks.

[1] Across the whole phylogeny using M0 model.
[2] Positive selection across the whole phylogeny.
[3] Positive selection on the branch between *Bombus* and *Apis*.
[4] Positive selection on the branch to *Bombus*.
[5] Positive selection on the branch to *Apis*.
[6] Positive selection on the branch to *Megachile*.
[7] Different selective pressures between *social* and *solitary*.

Using FDR < 0.05 on all tests.
